# The influence of urban environmental effects on the orchard soil microbial community structure and function: a case study in Zhejiang, China

**DOI:** 10.3389/fmicb.2024.1403443

**Published:** 2024-09-09

**Authors:** Rongchen Dai, Cuixiang Jin, Meng Xiao

**Affiliations:** ^1^Peking Union Medical College Hospital, Chinese Academy of Medical Sciences & Peking Union Medical College, Beijing, China; ^2^National Key Laboratory of Intelligent Tracking and Forecasting for Infectious Diseases, National Institute for Communicable Disease Control and Prevention, Chinese Center for Disease Control and Prevention, Beijing, China

**Keywords:** soil microbiota, ecological implications, soil health, environmental factors, rhizospheric zone

## Abstract

The urban environmental effects can have multifaceted impacts on the orchard soil microbial community structure and function. To specifically study these effects, we investigated the soil bacterial and fungal community in the laxly managed citrus orchards using amplicon sequencing. Ascomycota demonstrated significant dominance within the citrus orchard soils. The increased presence of beneficial *Trichoderma* spp. (0.3%) could help suppress plant pathogens, while the elevated abundance of potential pathogenic fungi, such as *Fusarium* spp. (0.4%), might raise the likelihood of disorders like root rot, thereby hindering plant growth and resulting in reduced yield. Moreover, we observed significant differences in the alpha and beta diversity of bacterial communities between urban and rural soils (*p* < 0.001). Environmental surveys and functional prediction of bacterial communities suggested that urban transportation factors and rural waste pollution were likely contributing to these disparities. When comparing bacterial species in urban and rural soils, *Bacillus* spp. exhibited notable increases in urban areas. *Bacillus* spp. possess heavy metal tolerance attributed to the presence of chromium reductase and nitroreductase enzymes involved in the chromium (VI) reduction pathway. Our findings have shed light on the intricate interplay of urban environmental effects and root systems, both of which exert influence on the soil microbiota. Apart from the removal of specific pollutants, the application of *Bacillus* spp. to alleviate traffic pollution, and the use of *Trichoderma* spp. for plant pathogen suppression were considered viable solutions. The knowledge acquired from this study can be employed to optimize agricultural practices, augment citrus productivity, and foster sustainable agriculture.

## 1 Introduction

Due to global climate change, microorganisms impacting human health and societal productivity underwent changes. This included the emergence of new pathogens (Casadevall, 2023), alterations in their growth and reproductive characteristics, particularly the increase in antibiotic resistance (Lockhart et al., 2023), the expansion of their distribution range, and seasonal fluctuations in microbial infectious diseases (Mora et al., 2022). The urban environment served as the primary habitat for modern human populations and played a crucial role in the interaction between humans and nature. For local regions, urban environmental effects such as the urban heat island effect and pollution emissions could have even more direct impacts on microorganisms than the effects of global climate warming (Parajuli et al., 2018; Abrego et al., 2020; Li et al., 2023). Toxic heavy metals, organic pollutants, emerging contaminants, and other biotic and abiotic stressors may impact nutrient utilization, plant metabolic pathways, agricultural productivity, and soil fertility (Pathak et al., 2024). Therefore, investigating the effects of urban environmental effects on microbial community structure could provide valuable insights into the distribution and transmission patterns of microorganisms and facilitate the assessment of environmental health risks (Blocker et al., 2020; Zhou and Zhou, 2023).

The soil environment serves as a reservoir for pathogenic microorganisms and is also a critical medium linking microbial communities and human activities (Williams et al., 2024; Wang B. et al., 2024; Wang X. et al., 2024). Pathogenic microorganisms in the soil not only directly impacted agricultural production but also resulted in alterations in the human microenvironment and even clinical infections (Liu et al., 2024; Singh et al., 2024; Yiallouris et al., 2024). In fact, soil microorganisms play crucial roles in shaping soil health (Cheng et al., 2019; Li et al., 2022), facilitating nutrient cycling (Hu et al., 2021), and fostering the overall health of the ecosystem (Tsitsigiannis et al., 2005). They participate in various biogeochemical processes, such as decomposition, nitrogen fixation (Pankievicz et al., 2021; Yin et al., 2021), and organic matter recycling (Yang et al., 2022). Moreover, microbes in rhizospheric soil engage in symbiotic interactions with plant roots (Matilla et al., 2007), influencing nutrient uptake (Wu et al., 2022), stress tolerance (Igiehon et al., 2021), and disease resistance (De Tender et al., 2021). Understanding the structure and function of microbial communities, particularly in the rhizospheric soil, is crucial for comprehending the distribution and transmission patterns of microorganisms, promoting agricultural production, and assessing the environmental health risks posed by urban effects (Aguilera et al., 2022; Furlan et al., 2023; Pastrana et al., 2023). Research on citrus has shown that soil microbial communities, especially those associated with the root system, significantly influence the quality of citrus fruits and interact with the host immune system (Su et al., 2023).

The warm and humid climate of Zhejiang Province, China, characterized by hot summers and mild winters, provides an ideal environment for the cultivation of citrus fruits, establishing citrus as a traditional fruit in the region (Lin et al., 2023). Given the lax soil management practices observed in this area, where active managerial intervention is infrequent, there emerges an opportunity to delve into the effects of urban environmental factors on soil microbiota within authentic field conditions. This was because the Zhejiang Province had a developed urban economy, with the primary, secondary, and tertiary industries accounting for 2.9, 41.9, and 55.2%, respectively (Jinhua City Statistics Bureau, 2023). Labor in rural areas tended to concentrate toward urban centers, leading plenty of older farmers to preferentially choose easily managed citrus for cultivation. Therefore, this study took citrus orchard soil environments close to their native state as examples. Soil samples were collected from the rhizospheric and peripheral soil at different distances from urban areas during the fruit ripening period on family farms. The microbial community structure and functional profiles in the soil were analyzed using sequencing methods targeting the 16S rRNA gene and ITS region gene.

The fruit ripening period is one of the most frequent times for farmers to interact with the soil and marks a critical phase in the life cycle of citrus trees, characterized by intricate physiological and ecological changes. Metabolism during this period is a key determinant of citrus flavor and nutritional quality (Saini et al., 2020; Sheng et al., 2022), and troubles during fruit ripening can lead to significant production losses (Olimi et al., 2022; Sharma et al., 2023; Kifle et al., 2024). Additionally, during this phase, the citrus orchard ecosystem experiences more frequent interactions, including proliferation of saprophytic microorganisms due to fruit rot and the presence of excreta from animals, primarily birds (Thompson and Willson, 1979; Schaefer and Ruxton, 2011; Whitehead and Poveda, 2011). Therefore, during this period, the impacts of urban environmental effects such as noise pollution and vehicular movement are more sensitively captured (Moore et al., 2002; Lopez-Velasco et al., 2012; Xi et al., 2016).

The objective of this study is to assess the impact of urban environmental effects on microbial distribution and environmental health risks through the compositional differences of microbes in the soil of citrus orchards in their native state during the fruit ripening period. Furthermore, the study aims to provide valuable evidence for research on environmental-host relationships, optimization of future agricultural production, and monitoring of the prevalence of pathogenic microorganisms using these research findings and microbial community data.

## 2 Materials and methods

### 2.1 Study site

The soil sampling was carried out in Jinhua, situated in the central region of Zhejiang Province, China. The sampling commenced on October 9, 2022, and continued for 10 days, with each sampling site’s process being completed within a single day. Jinhua is located approximately between 119°14′–120°47′ east longitude and 28°32′–29°41′ north latitude, spanning 129 km north to south and 151 km east to west. The orchard area covers 26,290 hectares, with 3,809 hectares dedicated to citrus orchards. This region falls within the subtropical zone, characterized by a warm and humid climate, with hot summers and mild winters. In 2022, the total annual sunshine hours were 1701.2, with a precipitation of 1300.3 mm and an average temperature of 19.4°C.

To ensure the similarity of sampling environments and eliminate other factors’ influence on soil microorganisms, we specifically selected family farms adjacent to rivers for environmental sampling. Four villages with lax management were chosen as sampling sites based on their geographic location. Lax management is defined as a management practice where, apart from essential watering, the intervention frequency (including pesticide and fertilizer usage) is equal to or less than twice a year; family farms with higher intervention frequencies were not considered. Among these, priority was given to farms that either abandoned management or had lower management frequencies.

**Figure 1:**
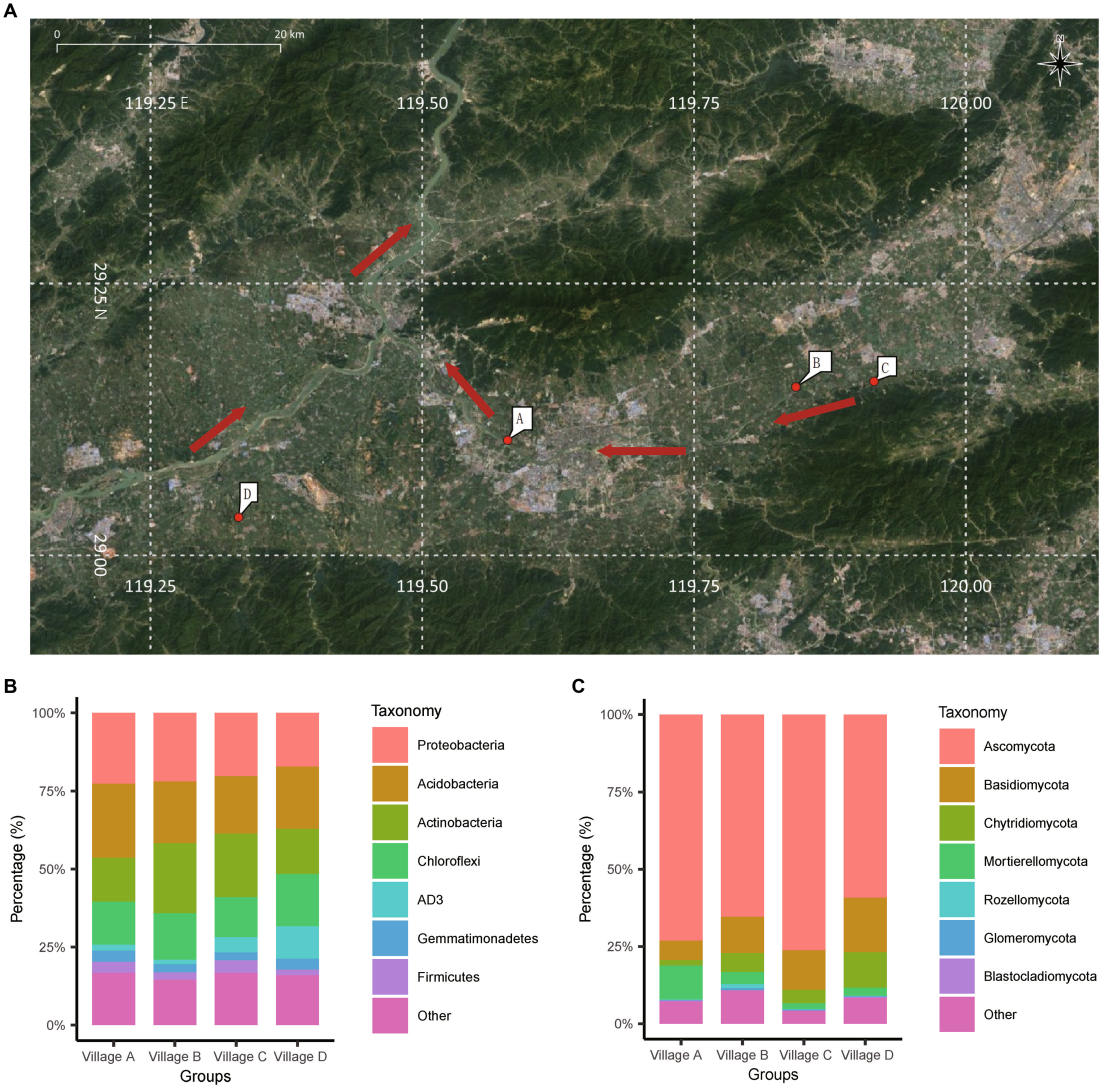
Geographic distribution and taxonomy annotation of collection sites. **(A)** The specific locations of the four villages in the soil sampling process are marked in the diagram. The red arrows in the image represent the direction of the river’s flow. **(B,C)** Stacked bar chart showing the average abundance of bacterial species **(B)** and fungal species **(C)** at the “phylum” level in the four villages. The chart highlights the top 8 bacterial species with the highest abundances. **(A)** Obtained from Google Satellite and annotated (https://www.google.com/maps/).

Village A located on the river downstream of the center of the city and closely adjacent to the city. It is situated in a densely populated area and is strongly influenced by urban population mobility. On the other hand, villages B, C, and D are situated farther from the city and are next to the river but upstream of the city (Figure 1A). Fourteen family farms were designated as individual sampling site (Supplementary Table S1), where five sampling points were selected: one sampling point was in the center of the farm, and one was at each corner. In order to assess disparities in composition of the soil in the rhizosphere and the soil outside of the rhizosphere, two additional sampling points were strategically placed outside the rhizosphere (peripheral soil). Specifically, these points were positioned at the midpoint between two fruit trees, where no root growth was observed.

### 2.2 Sample collection

We conducted sampling using five-point sampling method during the ripening stages of Jinhua’s native citrus. Rhizospheric soil samples were gathered from the rhizosphere, which was considered to be the area around the root system. Five citrus trees aged 8–10 years were selected at each sampling point, and soil samples were collected around the roots of each tree to a depth of approximately 20 cm. We did not sample soil from the roots of citrus trees showing signs of disease or root damage. For the two sampling points located at the midpoint between two fruit trees, peripheral soil samples were collected to the same depth (20 cm), but without including the rhizospheric soil. During soil excavation, areas within a 1-m radius of the soil surface containing evident contaminants and decayed matter were excluded.

Before sampling, we removed the topsoil using a shovel. For sampling of rhizospheric soil, we gently removed soil that was loosely attached to the roots. Every 10 mL of soil from the five locations within each sampling point was placed in separate sterile plastic bags and thoroughly mixed to create a homogenized composite sample. Approximately 15 mL of the homogenized soil was then transferred into a labeled centrifuge tube and stored at −20°C during transportation. Upon arrival at the laboratory, the samples were promptly transferred to a −80°C ultra-low temperature freezer for long-term storage. The detailed locations of the sampling points and relevant environmental information can be found in Supplementary Table S1.

Finally, we systematically collected a total of 98 soil samples from four villages. Within this dataset, villages A (urban), C (rural), and D (rural) were each represented by 21 samples. Village B (rural) contributed a comprehensive set of 35 soil samples due to its broader and more diverse citrus cultivation landscape.

### 2.3 Extraction of fungal and bacterial DNA

DNA extraction from soil samples was carried out using the QIAamp DNA Mini Kit (Qiagen, Hilden, Germany) following the manufacturer’s instructions. To assess the quality and concentration of the extracted DNA, a spectrophotometer (Nanodrop 2000; Thermo Fisher Scientific, Waltham, MA, United States) was employed. The extracted DNA was then stored at −80°C until PCR analysis.

### 2.4 PCR amplification, library preparation and sequencing

The primers used to amplify the ITS1 region and the 16S V3–V4 region gene were: ITS-1F, 5′-CTT GGT CAT TTA GAG GAA GTA A-3′ and ITS-2R, 5′-GCT GCG TTC TTC ATC GAT GC-3′; 16S-338F, 5′-ACT CCT ACG GGA GGC AGC AG-3′ and 16S-806R, 5′-GGACTACHVGGGTWTCTAAT-3′.

The first round of PCR amplification was performed using the following cycling conditions: 3 min at 95°C; 25 cycles of 30 s at 95°C, 30 s at 55°C, and 30 s at 72°C; and a final extension step at 72°C for 5 min. Each 25 μL PCR mixture contained 12.5 μL of 2× KAPA HiFi HotStart ReadyMix, 1 μL of each primer (1 μM), and 12.5 ng of template DNA. Post-PCR purification was achieved using AMPure XP beads.

The second round of PCR amplification was performed using the following cycling conditions: 3 min at 95°C; 8 cycles of 30 s at 95°C, 30 s at 55°C and 30 s at 72°C; and a final extension at 72°C for 5 min. Each 50 μL PCR mixture contained 25 μL of 2× KAPA HiFi HotStart ReadyMix, 5 μL of each primer, 10 μL of water, and 5 μL of template DNA from products purified in the previous step. A second round of AMPure XP beads purification was performed.

Following purification, the amplicons were pooled in equimolar proportions and subjected to paired-end sequencing on an Illumina MiSeq platform (Illumina, San Diego, United States), according to the standard protocol provided by Majorbio Bio-Pharm Technology Co. Ltd. The number of sequencing reads obtained can be found in Supplementary Table S2. The sequencing reads have been deposited in the NCBI BioProject under ID PRJNA1007597.

### 2.5 Bioinformatics

All raw data underwent filtering using Trimmomatic (Bolger et al., 2014) (version 0.39) to eliminate adapters, primers, and low-quality sequences; the parameters were SLIDINGWINDOW:4:15, LEADING:3, TRAILING:3, and MINLEN:80. Subsequently, the processed data were imported into the Quantitative Insights Into Microbial Ecology version 2 (QIIME2) pipeline (Bolyen et al., 2019) (version 2021.11.0) for quality control. The 16S rRNA gene sequences were merged using vsearch (Rognes et al., 2016) (version v2.15.0) and then denoised via deblur using default parameters (Amir et al., 2017) to generate representative sequences. The representative sequences of ITS region gene were inferred using default parameters with the DADA2 plugin (Callahan et al., 2016) plugin.

Taxonomy assignment was performed on all representative sequences after training the species classifier using the q2-feature-classifier (Bokulich et al., 2018) plugin. The training process involved utilizing primers to extract target sequences, followed by filtering out corresponding taxonomic information using the RESCRIPt (Robeson et al., 2021) plugin. Finally, the feature-classifier plugin was used for training. The results of taxonomic analyses indicated that 98.0% of the bacteria were successfully identified at the phylum level using the 16S rRNA gene, whereas only 67.6% of fungi were successfully identified. Visualization of the taxonomy results was carried out on QIIME2 View^1^. The amplicon package^2^ of R was used to illustrate species compositions and to create Venn diagrams and circle plots.

Following alignment of the representative sequences exhibiting high abundance using MAFFT (Katoh et al., 2019) (version 7.490), the construction of the maximum likelihood (ML) tree was performed using IQTREE (Minh et al., 2020) (version 2.2.0.3). To determine the best DNA model, the Edge-linked Partition Model (Chernomor et al., 2016) was employed, and branch supports were assessed through ultrafast bootstrap (Hoang et al., 2018).

Functional prediction of the 16S rRNA gene representative sequences was performed with the picrust2 (Douglas et al., 2020) plugin within the QIIME2 pipeline. For BugBase (Ward et al., 2017) phenotypic predictions, submissions were made via the online platform^3^. The Linear Discriminant Analysis Effect Size (LEfSe) algorithm was processed using the format2lefse function within the amplicon package of R, followed by submission to ImageGP (Keighley et al., 2022) for further analysis.

For functional classification of the ITS region gene, FUNGuild (Nguyen et al., 2016) was employed to parse fungal community datasets based on trophic mode, trait and growth form. Differences between two groups were performed with the Mann–Whitney *U* test.

### 2.6 Diversity and statistical analysis

Representative sequences were processed using the EasyAmplicon (Liu Y. X. et al., 2021) pipeline. Normalization was conducted using the “otutab_rare.R” script^4^ with default parameters. The parameter “--depth 0” was utilized to automatically determine the minimum rarefaction depth. The diversity plugin of QIIME2 was used to calculate alpha and beta diversity. Alpha diversity was assessed using the Abundance-based Coverage Estimator (ACE) index, and differences were compared using the Kruskal–Wallis test. Principal Co-ordinates Analysis (PCoA) based on Bray–Curtis distance was performed. The resulting data were visualized using the amplicon package and pheatmap package of R.^5^ Differential analysis was conducted using “EdgeR” and results were visualized by using the ‘compare_volcano.R’^6^, ‘compare_manhattan.sh’ (), and ‘compare_heatmap.sh’^7^ scripts to generate volcano plots, Manhattan plots, and comparative heatmaps, respectively. A significance threshold of *p* < 0.05 was considered for all statistical analyses.

## 3 Results

### 3.1 Structure and function of microbial communities in urban and rural citrus orchard soils

Among the successfully identified bacteria, the top seven abundant phyla were Proteobacteria, Acidobacteria, Actinobacteria, Chloroflexi, AD3, Gemmatimonadetes, and Firmicutes (Figure 1B). For fungi, the top seven abundant phyla were Ascomycota, Basidiomycota, Chytridiomycota, Mortierellomycota, Rozellomycota, Glomeromycota, and Blastocladiomycota. Notably, Ascomycota fungi were predominant across almost all of the soil samples, accounting for an average of 66.9% of the species (Figure 1C).

**Figure 2:**
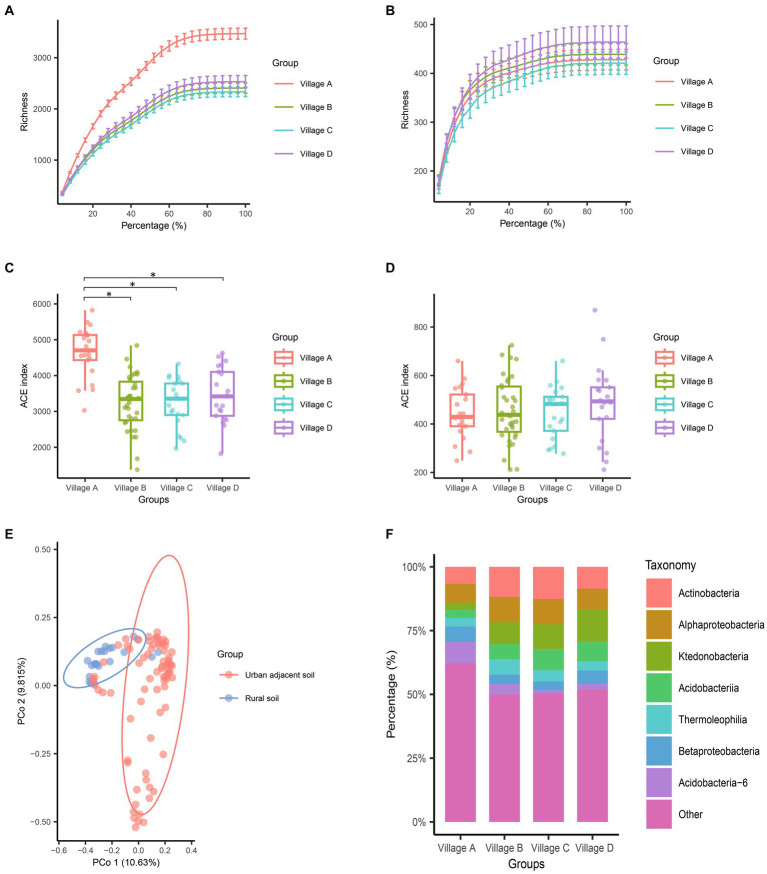
Comparison of species composition differences among different villages through alpha and beta diversity analysis. **(A,B)** Dilution curves of 16S **(A)** and ITS **(B)** sequences during the resampling process. **(C,D)** The alpha diversity differences of bacteria **(C)** and fungi **(D)** among the four villages were assessed using the Abundance-based Coverage Estimator (ACE) index. Each point in the box plot represents the ACE index of a sample. The lowercase letters “a” and “b” above the box plots indicate whether there is a statistical difference. The same letters indicate no statistical difference, while different letters indicate a statistical difference. **(E)** PCoA analysis based on Bray–Curtis distances between pairs of bacterial samples. The two dimensions with the highest explanatory power were plotted on the coordinate axes. **(F)** Stacked bar chart showing the average abundance of bacterial species at the class level in the four villages. The top 7 bacterial species with the highest abundances were highlighted. A *p*-value less than 0.05 was considered statistically significant.

Rarefaction curve analyses of the sequencing results demonstrated stable patterns across all samples. From the rarefaction curves, it appeared that village A, which is urban, might have a significantly higher bacterial abundance as compared to the other three villages, which are considered rural (Figure 2A). These intriguing insights were confirmed by comparisons of the alpha diversity values. With regard to pairwise comparisons of bacterial alpha diversity, no statistically significant differences were observed between villages B, C, and D. In contrast, pairwise comparisons of the bacterial alpha diversity values from villages A identified significant differences with the alpha diversity values from villages B (*p* < 0.001), C (*p* < 0.001), and D (*p* < 0.001) (Figure 2C and Table 1). We conducted PCoA analysis using pairwise comparisons of Bray–Curtis distances between samples. This analysis confirmed the significant bacterial dissimilarity between urban soil and rural soil (Figure 2E). Furthermore, at the phylum taxonomic level, a noticeable increase in species from Acidobacteria-6 and decreases in Actinobacteria and Ktedonobacteria species were observed in urban soil (Figure 2F).

**Table 1:** Kruskal–Wallis test for differential ACE index comparisons between the bacterial population in soil from four villages.

Group 1	Group 2	*H*	*p*-value	*q*-value
Village A (*n* = 21)	Village B (*n* = 35)	22.859	<0.001	<0.001
	Village C (*n* = 21)	19.713	<0.001	<0.001
	Village D (*n* = 21)	16.301	<0.001	<0.001
Village B (*n* = 35)	Village C (*n* = 21)	0.186	0.666	0.666
	Village D (*n* = 21)	0.447	0.504	0.605
Village C (*n* = 21)	Village D (*n* = 21)	0.753	0.385	0.578

Kruskal–Wallis (all groups), *H* = 29.446, *p*-value = 0.000001805.

The most prevalent fungal genera in the samples were *Talaromyces*, *Knufia*, *Fusarium*, *Chaetomium*, *Penicillium*, *Aspergillus*, *Paracamarosporium*, *Trichoderma*, and *Chaetomella*. In terms of fungal alpha diversity, there were no statistically significant differences observed among the villages collectively (*p* = 0.787) (Figures 2B,D). The results of PCoA analysis revealed only subtle differences between urban soil and rural soil (Supplementary Figure S1A), indicating a generally similar fungal community structure between these two soil environments.

Notable patterns emerged among pathways represented in rural soil compared to urban soil, with nicotine degradation II (pyrrolidine pathway) (*p* < 0.001), toluene degradation I (aerobic) (via o-cresol) (*p* < 0.001), toluene degradation II (aerobic) (via 4-methylcatechol) (*p* < 0.001), nylon-6 oligomer degradation (*p* = 0.007), aromatic compound degradation via *β*-ketoadipate (*p* < 0.001), and 4-coumarate degradation (anaerobic) (*p* < 0.001) exhibiting significant increases. Conversely, urban soil exhibited distinctive shifts compared to rural soil, with formaldehyde assimilation II (RuMP Cycle) (*p* = 0.016), formaldehyde oxidation I (*p* = 0.017), toluene degradation V (aerobic) (via toluene-cis-diol) (*p* = 0.002), and anaerobic aromatic compound degradation (*Thauera aromatica*) (*p* < 0.001) experiencing a marked increase in relative abundance (Supplementary Table S11).

### 3.2 Structure and function of microbial communities in rhizospheric and peripheral citrus orchard soils

**Figure 3:**
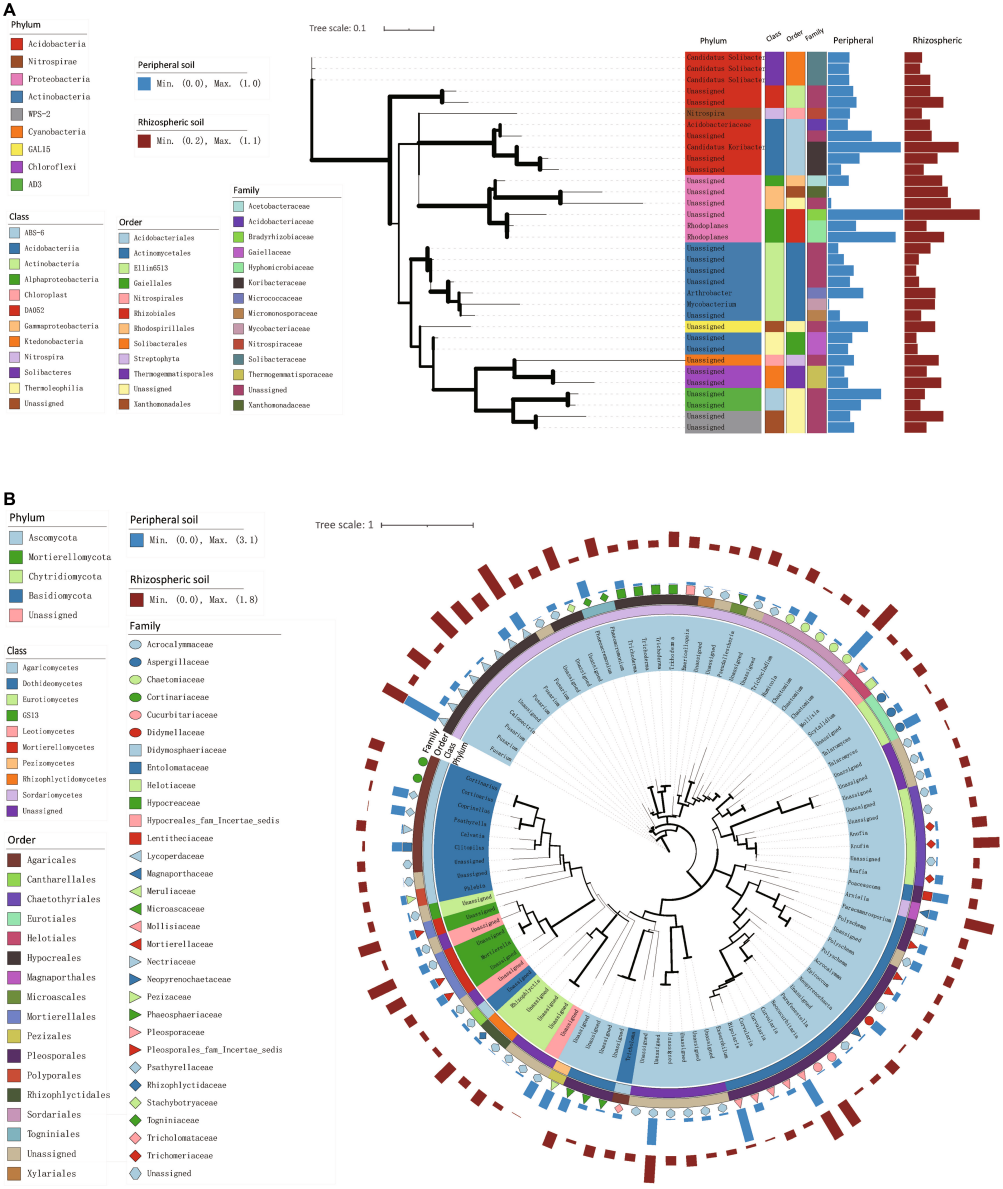
Phylogenetic trees of high-abundance species in rhizospheric and peripheral soil. **(A,B)** A maximum likelihood tree constructed from representative sequences of bacterial **(A)** and fungal **(B)** species with an average abundance proportion greater than 0.2% across all samples. The thickness of branches represents the magnitude of bootstrap values, and only values within the range of 0.2 to 1 are labeled.

In Figures 3A,B, we quantified highly abundant bacteria and fungi (with average relative abundances exceeding 0.2%) using maximum likelihood trees to depict the bacterial and fungal community structures and phylogenetic relationships within the rhizospheric and peripheral soils. This approach provided insights into the community composition and evolutionary connections of bacteria and fungi in these distinct soil environments.

**Figure 4:**
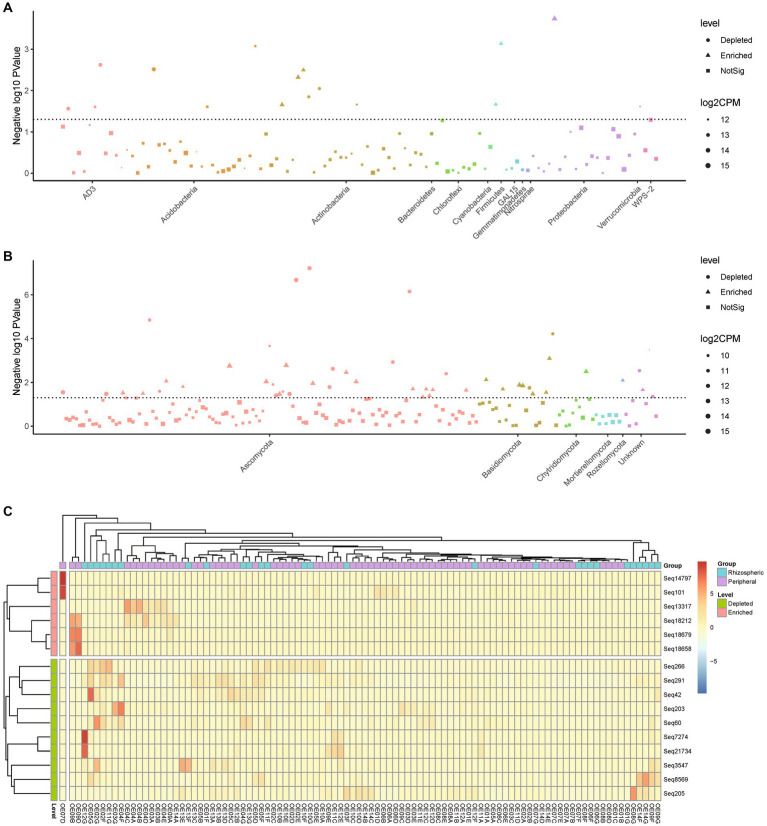
Comparative analysis of abundant differential species between rhizospheric and peripheral soil. **(A,B)** Manhattan plot depicting the statistically significant differences in the abundance proportions of bacterial **(A)** and fungal **(B)** species between rhizospheric soil and peripheral soil. Patterns above the dashed line indicate sequences with statistical differences. **(C)** Heatmap displaying statistically significant bacterial species’ differences between rhizospheric soil and peripheral soil, clustered by sample abundance. A *p*-value less than 0.05 was considered statistically significant.

Within the rhizospheric zone, noticeable increases were observed in six bacterial taxa, including Xanthomonadales, Bacilli, and Actinobacteria (Figures 4A,C). Similarly, 27 fungal taxa exhibited heightened representation, encompassing Agaricomycetes, Sordariomycetes, and Eurotiomycetes (Figure 4B). To further elucidate the effects of urban environmental factors and citrus root systems on soil microbiota, we classified samples simultaneously based on different sampling locations and soil types (Supplementary Figure S2). In villages B, C, and D, compared to peripheral soil, the alpha and beta diversity of rhizospheric soil exhibited greater fluctuations, the trend observed in both bacteria and fungi. Specifically, we observed that rhizospheric alpha diversity from the same village had larger interquartile ranges in boxplots compared to peripheral soil. Correspondingly, in the PCA plots of beta diversity, the confidence ellipsoids of rhizospheric samples from the same locations were more inclined to encompass those of peripheral soil. Conversely, in village A, the boxplots of alpha diversity for both bacteria and fungi indicated larger interquartile ranges in peripheral soil. Simultaneously, in the PCA plots, the confidence ellipsoids of peripheral soil and rhizosphere samples exhibited roughly equal intersection. These findings suggest that citrus root systems do indeed play a role in shaping soil microbial community structure, albeit weaker than the influence of urban environmental effects.

**Figure 5:**
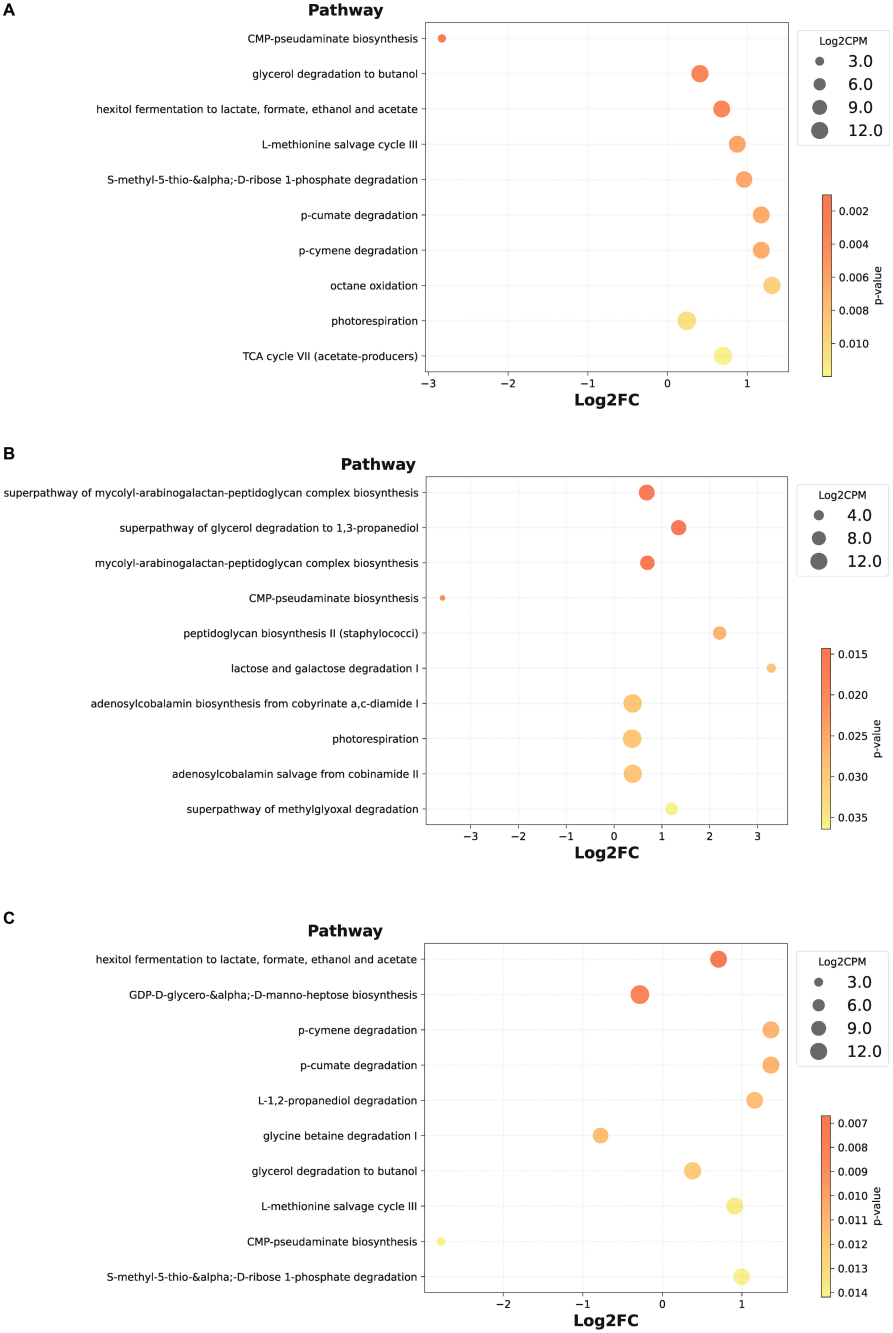
Differential bacterial species pathways in rhizospheric soil. **(A–C)** The bubble plot illustrates the differences in functional pathways of bacteria between rhizospheric soil vs. peripheral soil **(A)**, urban rhizospheric soil vs. urban peripheral soil **(B)**, rural rhizospheric soil vs. rural peripheral soil **(C)**, presenting the top 10 pathways with the smallest *p*-values in ascending order.

**Figure 6:**
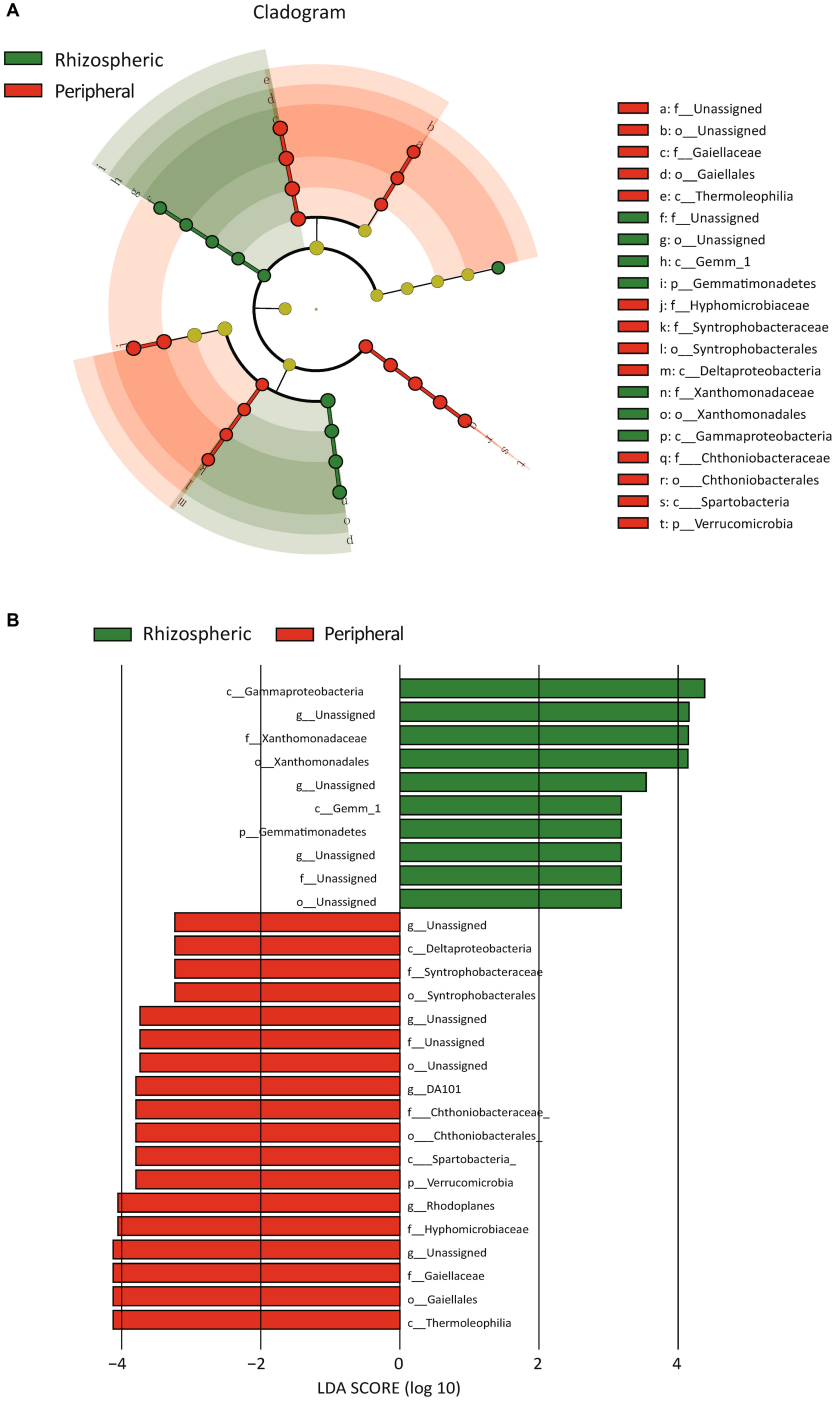
Bacterial distinctions in rhizospheric vs. peripheral soil revealed through LEfSe analysis. **(A)** The cladogram illustrates the microbial differences between rhizospheric soil and peripheral soil, highlighting only those microbial features that exhibit significant distinctions. Each circle represents a taxonomic level, such as phylum, class, and so forth. **(B)** The bar plot provides a detailed representation of the microbial differences between rhizospheric soil and peripheral soil.

Furthermore, we contrasted bacterial functional disparities between citrus orchard rhizospheric and peripheral soils (including both urban and rural), pinpointing a significant reduction in the CMP-pseudaminate biosynthesis pathway in the rhizospheric soil (*p* = 0.001) (Figure 5A). Importantly, the divergence of this pathway was unaffected by urban factors, as evidenced by its substantial reduction both urban rhizospheric soil as compared with urban peripheral soil (*p* = 0.020, Figure 5B) and in rural rhizospheric soil as compared with rural peripheral soil (*p* = 0.014, Figure 5C). The outcomes of a LEfSe indicated the potential prominence of Xanthomonadaceae within the rhizospheric zone (*p* = 0.007) (Figure 6). While functional differences were identified in bacterial communities in rhizospheric and peripheral soils, no statistically significant differences between the two soil types were identified using phenotype prediction across all of the following nine phenotype traits: aerobic, anaerobic, mobile element-containing, biofilm-forming, Gram-negative, Gram-positive, potentially pathogenic, and stress-tolerant (Supplementary Figures S3, S4).

The results from the FUNGuild analysis, which categorizes fungal communities based on trophic mode, traits, and growth forms, reveal noticeable differences between the fungal communities inhabiting the rhizospheric and peripheral zones of the soil. Specifically, in peripheral soils, the trophic mode classification identified a comparatively heightened prevalence of pathotrophs (*p* = 0.042), coupled with an increased occurrence of plant pathogens as per the Guild classification (*p* = 0.011). Additionally, analysis of the growth form classification demonstrated significantly elevated proportions of smut (*p* = 0.026) and corticioid (*p* = 0.020) (Table 2).

**Table 2:** Selected results with statistically significant differences from guild classification comparisons of the microbial populations of rhizospheric and peripheral soils.

	Species count	Peripheral soil (*n* = 28) (%)	Rhizospheric soil (*n* = 70) (%)	All (*n* = 98) (%)	*U* value	*p-*value
Trophic mode						
Pathotroph	556	10.932	6.145	7.512	1,239	0.042
Guild						
Plant pathogen	360	10.496	5.142	6.672	1,303	0.011
Animal pathogen	57	0.118	0.399	0.319	696.5	0.025
Endophyte-leaf saprotroph-plant pathogen	2	0.001	<0.001	<0.001	1,050	0.026
Endophyte-plant pathogen-wood saprotroph	11	0.244	0.192	0.207	1,243	0.036
Animal pathogen-fungal parasite-undefined saprotroph	29	0.077	0.585	0.440	682	0.015
Animal pathogen-endophyte-epiphyte-fungal parasite-plant pathogen-wood Saprotroph	53	1.106	2.296	1.956	676	0.017
Animal pathogen-plant pathogen-undefined saprotroph	67	1.254	0.812	0.938	1,238	0.043
Endophyte-fungal parasite-lichen parasite-plant pathogen-wood saprotroph	2	1.339	0.010	0.454	1,278	0.016
Growth form						
Smut	2	0.001	<0.001	<0.001	1,050	0.026
Corticioid	86	0.741	0.48	0.561	1271.5	0.020

## 4 Discussion

### 4.1 Ascomycota fungal dominance in citrus orchard soils: ecological and agricultural implications

Ascomycota exhibited a substantial dominance within the citrus orchard soils. This pronounced presence of a specific taxon likely exerted a multifaceted impact on both the ecological landscape of citrus orchard soils and the agricultural productivity of the area. For example, the elevated prevalence of beneficial *Trichoderma* spp. (0.3%) would suppress plant pathogens. The principal mechanisms not only include mycoparasitism, antibiosis, and competition for resources and space but also involve the induction of resistance pathways in plants, leading to increased plant growth and nutrient uptake. Additionally, these fungi produce a diverse array of antifungal enzymes, such as chitinases and beta-1,3 glucanases. These enzymes exhibit synergistic effects with each other and with other antifungal enzymes and materials (Harman, 2006; Bae et al., 2011). Finally, rhizosphere-competent *T. harzianum* strain, such as T22, have been shown to promote root growth in a variety of plants (Harman, 2000). Conversely, the heightened abundance of potential pathogenic fungi, such as *Fusarium* spp. (0.4%), might increase the risk of disorders like root rot, consequently impeding plant growth and leading to diminished yield (Buttar et al., 2024; Kamble et al., 2024).

In terms of fruit quality, the prominent occurrence of *Penicillium* (0.4%) and *Aspergillus* spp. (0.4%). might lead to surface mold formation on citrus fruits, thereby influencing their visual appearance and gastronomic value. Moreover, certain strains within the *Penicillium* spp. could generate deleterious toxins, thereby posing latent risks to human health. Additionally, the augmented abundance of fungi similar to *Chaetomium* (0.4%) and *Chaetomella* spp. (0.3%), in turn, could substantively contribute to organic matter decomposition.

In the realm of farm management, a comprehensive understanding of the ecological roles of each species remains paramount. This comprehension is instrumental in devising judicious soil management strategies and disease control measures, designed to harness the proactive potential of beneficial species while mitigating the detrimental effects of harmful ones.

### 4.2 Deciphering urban–rural influences on citrus orchard soil: comprehensive analysis from diversity disparities to functional pathway differences

In the realm of diversity analysis, distinct differences were evident in the alpha diversity of bacterial communities between the citrus orchard soil of village A and those of villages B (*p* < 0.001), C (*p* < 0.001), and D (*p* < 0.001). The substantial disparity in species diversity became apparent through analyses of rarefaction curve plots, beta diversity plots, and taxonomic classifications at the phylum level (Figure 1). Considering the alignment of sampling design and real-world environmental observations, we inferred that the proximity to urban transportation zones played a pivotal role in driving differences in soil bacterial diversity between urban soil and rural soils. This inference was corroborated by the results of the functional disparity analysis of bacterial communities between urban soil and rural soil.

In the urban soil, the significant increase in the relative abundance of Formaldehyde assimilation II (RuMP Cycle) and formaldehyde oxidation I possibly stems from pollution sources, including vehicular emissions near urban areas, prompting bacterial communities to adapt metabolically to formaldehyde (Klein et al., 2022). Similarly, the notable increase in the relative abundance of toluene degradation V (aerobic) (via toluene-cis-diol) might be attributed to the prevalence volatile organic compounds, such as toluene, in urban environments, leading to metabolic adaptation among bacterial communities (Gelencsér et al., 1997). The significant rise in the relative abundance of anaerobic aromatic compound degradation (*Thauera aromatica*) is consistent with the presence of substantial aromatic compounds in the urban environment and the adaptation of bacterial communities to degrade these compounds (Keyte et al., 2016; Zhang et al., 2022).

The urban orchards were closely situated alongside a motorized road, resulting in heightened vehicular activity during routine periods. Research on the impact of highway-related activities on soil microorganisms has shown that these activities exacerbate vegetation degradation along the road, significantly alter soil physicochemical properties, and cause heavy metal pollution, thereby affecting the diversity and community structure of soil bacteria. Furthermore, this disturbance exhibits a gradual increase in intensity with proximity to the highway (Liu Z. et al., 2021). In comparison of bacterial species in village A (urban) and the other villages (rural), *Bacillus muralis*, *Bacillus fumarioli*, and *Bacillus flexus* all showed significant increases in urban. *Bacillus* spp. possess heavy metal tolerance due to the presence of chromium reductase and nitroreductase enzymes, which are involved in the chromium (VI) reduction pathway (Luo et al., 2022; Shahraki et al., 2022; Ramli et al., 2023). This finding suggests that the changes in soil microbial community structure observed are influenced by traffic factors, consistent with the environmental factors and research results we have observed. It also suggests that in the study area, the application of *B. muralis*, *B. fumarioli*, or *B. flexus* for soil microbial remediation could be considered (Jebeli et al., 2017; Kulkova et al., 2023; Schommer et al., 2023; Yu et al., 2023).

Litter can increase the network complexity of soil microbial communities (Feng et al., 2022). Changes in litter and roots can affect soil microbial activities and nutrient cycling (Chen et al., 2022; Zhu et al., 2022). Potentially important physical differences between rural and urban areas were noticed during soil collection. We observed dispersed waste materials in rural orchards. These materials were primarily composed of discarded cigarette remnants, abandoned bags, garments, and packaging materials (Supplementary Figure S5), whereas similar materials were not observed in the urban orchards. Correspondingly, there was a significant increase in the relative abundance of bacteria involved in nicotine degradation II (pyrrolidine pathway), indicating a potential bacterial metabolic adaptation to nicotine degradation. The significant elevation in the relative abundance of nylon-6 oligomer degradation might be linked to waste materials prevalent in rural environments.

Fruit tree root system can affect soil microbial community structure, but it was not the main factor. According to our analysis, we observed a significant difference (*p* = 0.001) in the CMP-pseudaminate biosynthesis pathway between the rhizospheric soil and the peripheral soil (Figure 5). Pseudaminic acid, a sialic acid-like sugar, is involved in the synthesis of bacterial cell surface glycans, which in turn affects pathogenicity and adaptability through bacterial adhesion, invasion, and immune evasion mechanisms (Knirel et al., 2003; Hsu et al., 2006). The reduction in the CMP-pseudaminate biosynthesis pathway may represent a plant-microbe interaction, where plant roots release certain compounds that influence bacterial metabolism and biosynthetic pathways in the rhizospheric soil, contributing to a protective effect. However, it is important to note that our study also revealed a relatively high false discovery rate (FDR = 0.415, Supplementary Table S10), which may indicate a certain degree of false positives in multiple comparisons. Further research is needed to validate these findings and explore the biological significance of the observed differences in more depth.

These variations in bacterial functional pathways support our hypotheses regarding the impact of urban environmental factors on citrus orchard soil. The exogenous factors such as urban traffic and rural litter did have a significant impact on the microbiome in citrus orchards. These factors played a significant role in the substantial differences in bacterial diversity, potentially further influencing citrus tree growth and fruit yield.

### 4.3 Implementing practical strategies for citrus orchard soil management: insights and recommendations

By elucidating the microbial dynamics in citrus orchard soil during the crucial fruit maturation period, our findings can inform management strategies aimed at enhancing citrus yield and fruit quality. The identification of beneficial soil microorganisms capable of promoting citrus growth and disease resistance can lead to the development of targeted microbial-based biofertilizers and biocontrol strategies (Scott et al., 2006; Das et al., 2022; Wang et al., 2023), reducing reliance on chemical inputs and fostering long-term sustainability (Ali et al., 2019; Li et al., 2019; Zhou et al., 2020).

We propose the implementation of physical barriers in rural areas to reduce the quantity of pollutants such as cigarette butts and nylon. Urban orchards in city areas should be located far away from densely populated regions with high traffic density (Hagler et al., 2012; Ranasinghe et al., 2019). However, considering the current pollution situation, microbial measures can also be employed for improvement. For example, the *Trichoderma harzianum* T22 strain can be cultured on potato dextrose agar medium, and the spores can be collected for seed coating (Harman and Shoresh, 2007; Alinc et al., 2021). Alternatively, soaking the seeds in a suspension containing *Bacillus* spp. for 2 h can help improve plant growth and crop productivity under various abiotic stresses, including heavy metals and drought (Anbuganesan et al., 2024). In addition, synthesizing a biodegradable carbon nanoparticle from bacterial biofilm is also an option (Li et al., 2024; Savadiya et al., 2024). A more direct approach is to consider inserting genes encoding antifungal protein internal chitinase or external chitinase into the citrus genome alone or in combination (Bolar et al., 2000, 2001).

By utilizing amplicon sequencing as a non-invasive and cost-effective method, we were able to gain insights into the microbial diversity and functional potential in citrus orchard soil at an unprecedented scale. Our study confirmed the beneficial application of metagenomics in addressing agricultural and environmental concerns. This included the identification of two pollutants that indeed affected microbial community functionality and proposed specific microbial methods for improving land heavy metal pollution and suppressing plant pathogens based on real-world microbial community structures. Consequently, future research could continue to utilize amplicon sequencing across different seasons and larger geographical scales, and shotgun sequencing methods could be employed to further validate the functionality of these microbes.

While our study has provided valuable insights into the microbial communities of citrus orchard soils, several limitations need to be considered. Firstly, our research solely relied on functional prediction methods to examine microbial community functional changes, which resulted in a lack of sufficient validation and interpretability. Future studies should incorporate more comprehensive functional analyses, such as employing shotgun sequencing methods to investigate specific differentially expressed genes. Moreover, our study did not include intervention studies using specific species, which could have strengthened the credibility of the proposed measures for soil health and agricultural productivity. Additionally, although we consider the fruit maturation stage as a crucial phase for investigation, it is essential to study soil in other growth periods to ensure the stability assessment of our findings. To maximize the comparability of sampling points, we applied specific restrictions, focusing on citrus orchards situated near rivers and lacking human management. Consequently, in urban settings, other suitable family farms meeting these criteria were not found. Further urban sampling and exploration across other soil environment categories are essential to enhance the universality of our conclusions. Finally, our study lacked a comprehensive assessment of environmental factors, which may have led to the omission of certain variables. Addressing these limitations in future research could further enhance our understanding of microbial communities in citrus orchard soils.

## 5 Conclusion

In this study, we aimed to elucidate the microbial community structures within the rhizospheric zone and peripheral soils of citrus orchards by employing environmental sampling and amplicon sequencing techniques. Our findings have shed light on the intricate interplay of urban environmental effects and root systems, both of which exert influence on the soil microbiota. Apart from the removal of specific pollutants, the application of *B. muralis*, *B. fumarioli*, and *B. flexus* to alleviate traffic pollution, and the use of *Trichoderma* spp. for plant pathogen suppression were considered viable solutions. The knowledge acquired from this study can be employed to optimize agricultural practices, augment citrus productivity, and foster sustainable agriculture in Zhejiang Province and beyond China.

Overall, our study, utilizing amplicon sequencing methods, identified that urban and rural environmental effects indeed influenced the microbial community structure in citrus orchard soils. A valuable approach for enhancing the stability and feasibility of conclusions in the future would involve the long-term observation of environmental and agricultural issues across various stages of plant growth using metagenomic methods.

## Data availability statement

All sequencing data have been deposited in the NCBI database (BioProject No. PRJNA1007597).

## Author contributions

RD: Conceptualization, Data curation, Formal analysis, Investigation, Methodology, Project administration, Software, Supervision, Validation, Visualization, Writing – original draft, Writing – review & editing, Resources. CJ: Data curation, Investigation, Methodology, Project administration, Resources, Supervision, Validation, Writing – original draft, Writing – review & editing. MX: Conceptualization, Funding acquisition, Project administration, Resources, Supervision, Validation, Visualization, Writing – review & editing.

## Footnotes

^1^view.qiime2.org

^2^github.com/microbiota/amplicon

^3^bugbase.cs.umn.edu/upload.html

^4^github.com/YongxinLiu/EasyMicrobiome/blob/master/script/otutab_rare.R

^5^github.com/cran/pheatmap

^6^github.com/YongxinLiu/EasyMicrobiome/blob/master/script/compare_volcano.R

^7^github.com/YongxinLiu/EasyMicrobiome/blob/master/script/compare_heatmap.sh

## References

[ref1] Abrego N. Crosier B. Somervuo P. Ivanova N. Abrahamyan A. Abdi A.. (2020). Fungal communities decline with urbanization-more in air than in soil. ISME J. 14, 2806–2815. doi: 10.1038/s41396-020-0732-1, PMID: 32759974 PMC7784924

[ref2] Aguilera P. Becerra N. Alvear M. Ortiz N. Turrini A. Azcon-Aguilar C.. (2022). Arbuscular mycorrhizal fungi from acidic soils favors production of tomatoes and lycopene concentration. J. Sci. Food Agric. 102, 2352–2358. doi: 10.1002/jsfa.11573, PMID: 34636032

[ref3] Ali A. Ghani M. I. Ding H. Fan Y. Cheng Z. Iqbal M. (2019). Co-amended synergistic interactions between *Arbuscular mycorrhizal* Fungi and the organic substrate-induced cucumber yield and fruit quality associated with the regulation of the AM-fungal community structure under anthropogenic cultivated soil. Int. J. Mol. Sci. 20:1539. doi: 10.3390/ijms20071539, PMID: 30934751 PMC6479614

[ref4] Alinc T. Cusumano A. Peri E. Torta L. Colazza S. (2021). Trichoderma harzianum strain T22 modulates direct defense of tomato plants in response to *Nezara viridula* feeding activity. J. Chem. Ecol. 47, 455–462. doi: 10.1007/s10886-021-01260-3, PMID: 33713251 PMC8116274

[ref5] Amir A. McDonald D. Navas-Molina J. A. Kopylova E. Morton J. T. Zech Xu Z.. (2017). Deblur rapidly resolves single-nucleotide community sequence patterns. mSystems 2:e00191-16. doi: 10.1128/mSystems.00191-16 28289731 PMC5340863

[ref6] Anbuganesan V. Vishnupradeep R. Bruno L. B. Sharmila K. Freitas H. Rajkumar M. (2024). Combined application of biochar and plant growth-promoting Rhizobacteria improves heavy metal and drought stress tolerance in *Zea mays*. Plants 13:1143. doi: 10.3390/plants13081143, PMID: 38674552 PMC11053748

[ref7] Bae H. Roberts D. P. Lim H. S. Strem M. D. Park S. C. Ryu C. M.. (2011). Endophytic Trichoderma isolates from tropical environments delay disease onset and induce resistance against Phytophthora capsici in hot pepper using multiple mechanisms. Mol. Plant-Microbe Interact. 24, 336–351. doi: 10.1094/MPMI-09-10-0221 21091159

[ref8] Blocker L. Watson C. Wichern F. (2020). Living in the plastic age – different short-term microbial response to microplastics addition to arable soils with contrasting soil organic matter content and farm management legacy. Environ. Pollut. 267:115468. doi: 10.1016/j.envpol.2020.115468, PMID: 32891047

[ref9] Bokulich N. A. Kaehler B. D. Rideout J. R. Dillon M. Bolyen E. Knight R.. (2018). Optimizing taxonomic classification of marker-gene amplicon sequences with QIIME 2's q2-feature-classifier plugin. Microbiome 6:90. doi: 10.1186/s40168-018-0470-z, PMID: 29773078 PMC5956843

[ref10] Bolar J. P. Norelli J. L. Harman G. E. Brown S. K. Aldwinckle H. S. (2001). Synergistic activity of endochitinase and exochitinase from Trichoderma atroviride (*T. harzianum*) against the pathogenic fungus (*Venturia inaequalis*) in transgenic apple plants. Transgenic Res. 10, 533–543. doi: 10.1023/a:1013036732691, PMID: 11817541

[ref11] Bolar J. P. Norelli J. L. Wong K. W. Hayes C. K. Harman G. E. Aldwinckle H. S. (2000). Expression of Endochitinase from Trichoderma harzianum in transgenic apple increases resistance to apple scab and reduces vigor. Phytopathology 90, 72–77. doi: 10.1094/PHYTO.2000.90.1.72 18944574

[ref12] Bolger A. M. Lohse M. Usadel B. (2014). Trimmomatic: a flexible trimmer for Illumina sequence data. Bioinformatics 30, 2114–2120. doi: 10.1093/bioinformatics/btu170, PMID: 24695404 PMC4103590

[ref13] Bolyen E. Rideout J. R. Dillon M. R. Bokulich N. A. Abnet C. C. Al-Ghalith G. A.. (2019). Reproducible, interactive, scalable and extensible microbiome data science using QIIME 2. Nat. Biotechnol. 37, 852–857. doi: 10.1038/s41587-019-0209-9, PMID: 31341288 PMC7015180

[ref14] Buttar Z. A. Cheng M. Wei P. Zhang Z. Lv C. Zhu C.. (2024). Update on the basic understanding of *Fusarium graminearum* virulence factors in common wheat research. Plants 13:1159. doi: 10.3390/plants13081159, PMID: 38674569 PMC11053692

[ref15] Callahan B. J. McMurdie P. J. Rosen M. J. Han A. W. Johnson A. J. Holmes S. P. (2016). DADA2: high-resolution sample inference from Illumina amplicon data. Nat. Methods 13, 581–583. doi: 10.1038/nmeth.3869, PMID: 27214047 PMC4927377

[ref16] Casadevall A. (2023). Global warming could drive the emergence of new fungal pathogens. Nat. Microbiol. 8, 2217–2219. doi: 10.1038/s41564-023-01512-w 38030896

[ref17] Chen J. He J. Zhang Y. Huang J. Chen Z. Zeng W.. (2022). Effects of tobacco plant residue return on rhizosphere soil microbial community. Ann. Microbiol. 72:42. doi: 10.1186/s13213-022-01699-z

[ref18] Cheng Y. T. Zhang L. He S. Y. (2019). Plant-microbe interactions facing environmental challenge. Cell Host Microbe 26, 183–192. doi: 10.1016/j.chom.2019.07.009, PMID: 31415751 PMC6697056

[ref19] Chernomor O. von Haeseler A. Minh B. Q. (2016). Terrace aware data structure for phylogenomic inference from supermatrices. Syst. Biol. 65, 997–1008. doi: 10.1093/sysbio/syw037, PMID: 27121966 PMC5066062

[ref20] Das D. Ullah H. Himanshu S. K. Tisarum R. Cha-Um S. Datta A. (2022). Arbuscular mycorrhizal fungi inoculation and phosphorus application improve growth, physiological traits, and grain yield of rice under alternate wetting and drying irrigation. J. Plant Physiol. 278:153829. doi: 10.1016/j.jplph.2022.153829, PMID: 36202058

[ref21] De Tender C. Vandecasteele B. Verstraeten B. Ommeslag S. De Meyer T. De Visscher J.. (2021). Chitin in strawberry cultivation: foliar growth and defense response promotion, but reduced fruit yield and disease resistance by nutrient imbalances. Mol. Plant-Microbe Interact. 34, 227–239. doi: 10.1094/MPMI-08-20-0223-R 33135964

[ref22] Douglas G. M. Maffei V. J. Zaneveld J. R. Yurgel S. N. Brown J. R. Taylor C. M.. (2020). PICRUSt2 for prediction of metagenome functions. Nat. Biotechnol. 38, 685–688. doi: 10.1038/s41587-020-0548-6, PMID: 32483366 PMC7365738

[ref23] Feng J. Li Z. Hao Y. Wang J. Ru J. Song J.. (2022). Litter removal exerts greater effects on soil microbial community than understory removal in a subtropical-warm temperate climate transitional forest. For. Ecol. Manag. 505:119867. doi: 10.1016/j.foreco.2021.119867

[ref24] Furlan J. P. R. Sellera F. P. Stehling E. G. (2023). Strengthening genomic surveillance of carbapenemases in soils: a call for global attention. Lancet Microbe 4, e386–e387. doi: 10.1016/S2666-5247(23)00093-9, PMID: 36966797

[ref25] Gelencsér A. Siszler K. Hlavay J. (1997). Toluene−benzene concentration ratio as a tool for characterizing the distance from vehicular emission sources. Environ. Sci. Technol. 31, 2869–2872. doi: 10.1021/es970004c

[ref26] Hagler G. S. Lin M. Y. Khlystov A. Baldauf R. W. Isakov V. Faircloth J.. (2012). Field investigation of roadside vegetative and structural barrier impact on near-road ultrafine particle concentrations under a variety of wind conditions. Sci. Total Environ. 419, 7–15. doi: 10.1016/j.scitotenv.2011.12.002, PMID: 22281040

[ref27] Harman G. E. (2000). Myths and dogmas of biocontrol changes in perceptions derived from research on *Trichoderma harzianum* T-22. Plant Dis. 84, 377–393. doi: 10.1094/PDIS.2000.84.4.377, PMID: 30841158

[ref28] Harman G. E. (2006). Overview of mechanisms and uses of *Trichoderma* spp. Phytopathology 96, 190–194. doi: 10.1094/PHYTO-96-0190, PMID: 18943924

[ref29] Harman G.E. Shoresh M. (2007). The mechanisms and applications of symbiotic opportunistic plant symbionts, in: Novel biotechnologies for biocontrol agent enhancement and management, eds. Vurro M. Gressel J.: Springer Netherlands), 131–155.

[ref30] Hoang D. T. Chernomor O. von Haeseler A. Minh B. Q. Vinh L. S. (2018). UFBoot2: improving the ultrafast bootstrap approximation. Mol. Biol. Evol. 35, 518–522. doi: 10.1093/molbev/msx281, PMID: 29077904 PMC5850222

[ref31] Hsu K. L. Pilobello K. T. Mahal L. K. (2006). Analyzing the dynamic bacterial glycome with a lectin microarray approach. Nat. Chem. Biol. 2, 153–157. doi: 10.1038/nchembio767, PMID: 16462751

[ref32] Hu D. Baskin J. M. Baskin C. C. Liu R. Yang X. Huang Z. (2021). A seed mucilage-degrading fungus from the rhizosphere strengthens the plant-soil-microbe continuum and potentially regulates root nutrients of a Cold Desert shrub. Mol. Plant-Microbe Interact. 34, 538–546. doi: 10.1094/MPMI-01-21-0014-FI, PMID: 33596107

[ref33] Igiehon N. O. Babalola O. O. Cheseto X. Torto B. (2021). Effects of rhizobia and arbuscular mycorrhizal fungi on yield, size distribution and fatty acid of soybean seeds grown under drought stress. Microbiol. Res. 242:126640. doi: 10.1016/j.micres.2020.126640, PMID: 33223380

[ref34] Jebeli M. A. Maleki A. Amoozegar M. A. Kalantar E. Izanloo H. Gharibi F. (2017). *Bacillus flexus* strain as-12, a new arsenic transformer bacterium isolated from contaminated water resources. Chemosphere 169, 636–641. doi: 10.1016/j.chemosphere.2016.11.129, PMID: 27912188

[ref35] Jinhua City Statistics Bureau (2023). Jinhua municipal Bureau of Statistics 2023 statistical yearbook. Available at: http://tjj.jinhua.gov.cn/index.html

[ref36] Kamble A. Michavila S. Gimenez-Ibanez S. Redkar A. (2024). Shared infection strategy of a fungal pathogen across diverse lineages of land plants, the *Fusarium* example. Curr. Opin. Plant Biol. 77:102498. doi: 10.1016/j.pbi.2023.102498, PMID: 38142620

[ref37] Katoh K. Rozewicki J. Yamada K. D. (2019). MAFFT online service: multiple sequence alignment, interactive sequence choice and visualization. Brief. Bioinform. 20, 1160–1166. doi: 10.1093/bib/bbx108, PMID: 28968734 PMC6781576

[ref38] Keighley C. Gall M. van Hal S. J. Halliday C. L. Chai L. Y. A. Chew K. L.. (2022). Whole genome sequencing shows genetic diversity, as well as clonal complex and gene polymorphisms associated with fluconazole non-susceptible isolates of *Candida tropicalis*. J Fungi 8:896. doi: 10.3390/jof8090896, PMID: 36135621 PMC9505729

[ref39] Keyte I. J. Albinet A. Harrison R. M. (2016). On-road traffic emissions of polycyclic aromatic hydrocarbons and their oxy- and nitro- derivative compounds measured in road tunnel environments. Sci. Total Environ. 566-567, 1131–1142. doi: 10.1016/j.scitotenv.2016.05.152, PMID: 27312273

[ref40] Kifle D. R. Bacha K. B. Hora R. N. Likasa L. L. (2024). Evaluation of microbiome and physico-chemical profiles of fresh fruits of *Musa paradisiaca*, *Citrus sinensis* and *Carica papaya* at different ripening stages: implication to quality and safety management. PLoS One 19:e0297574. doi: 10.1371/journal.pone.0297574, PMID: 38289915 PMC10826968

[ref41] Klein V. J. Irla M. Gil Lopez M. Brautaset T. Fernandes Brito L. (2022). Unravelling formaldehyde metabolism in Bacteria: road towards synthetic Methylotrophy. Microorganisms 10:220. doi: 10.3390/microorganisms10020220, PMID: 35208673 PMC8879981

[ref42] Knirel Y. A. Shashkov A. S. Tsvetkov Y. E. Jansson P. E. Zahringer U. (2003). 5,7-diamino-3,5,7,9-tetradeoxynon-2-ulosonic acids in bacterial glycopolymers: chemistry and biochemistry. Adv. Carbohydr. Chem. Biochem. 58, 371–417. doi: 10.1016/s0065-2318(03)58007-6 14719362

[ref43] Kulkova I. Dobrzynski J. Kowalczyk P. Belzecki G. Kramkowski K. (2023). Plant growth promotion using *Bacillus cereus*. Int. J. Mol. Sci. 24:759. doi: 10.3390/ijms24119759, PMID: 37298706 PMC10253305

[ref44] Li M. Chen L. Zhao F. Tang J. Bu Q. Wang X.. (2023). Effects of urban–rural environmental gradient on soil microbial Community in Rapidly Urbanizing Area. Ecosyst. Health Sustain. 9:0118. doi: 10.34133/ehs.0118

[ref45] Li X. Fan J. Zhu F. Yan Z. Hartley W. Yang X.. (2024). Sb/as immobilization and soil function improvement under the combined remediation strategy of modified biochar and Sb-oxidizing bacteria at a smelting site. J. Hazard. Mater. 471:134302. doi: 10.1016/j.jhazmat.2024.134302, PMID: 38640664

[ref46] Li Z. Gao L. Chang P. Chen Z. Zhang X. Yin W.. (2022). The impact of *Elsinoe ampelina* infection on key metabolic properties in *Vitis vinifera* 'Red Globe' berries via multiomics approaches. Mol. Plant-Microbe Interact. 35, 15–27. doi: 10.1094/MPMI-09-21-0225-R, PMID: 34533970

[ref47] Li R. Pan X. Tao Y. Jiang D. Chen Z. Dong F.. (2019). Systematic evaluation of chiral fungicide Imazalil and its major metabolite R14821 (Imazalil-M): stability of enantiomers, enantioselective bioactivity, aquatic toxicity, and dissipation in greenhouse vegetables and soil. J. Agric. Food Chem. 67, 11331–11339. doi: 10.1021/acs.jafc.9b03848, PMID: 31529945

[ref48] Lin M. Xu C. Gao X. Zhang W. Yao Z. Wang T.. (2023). Comparative study on secondary metabolites from different citrus varieties in the production area of Zhejiang. Front. Nutr. 10:1159676. doi: 10.3389/fnut.2023.1159676, PMID: 37252230 PMC10211264

[ref49] Liu Y. X. Qin Y. Chen T. Lu M. Qian X. Guo X.. (2021). A practical guide to amplicon and metagenomic analysis of microbiome data. Protein Cell 12, 315–330. doi: 10.1007/s13238-020-00724-8, PMID: 32394199 PMC8106563

[ref50] Liu Z. Yang Y. Ji S. Dong D. Li Y. Wang M.. (2021). Effects of elevation and distance from highway on the abundance and community structure of Bacteria in soil along Qinghai-Tibet highway. Int. J. Environ. Res. Public Health 18:137. doi: 10.3390/ijerph182413137, PMID: 34948747 PMC8701971

[ref51] Liu Z. Zhang J. Fan C. Sun S. An X. Sun Y.. (2024). Influence of *Bacillus subtilis* strain Z-14 on microbial ecology of cucumber rhizospheric vermiculite infested with *Fusarium oxysporum* f. sp. *cucumerinum*. Pestic. Biochem. Physiol. 201:105875. doi: 10.1016/j.pestbp.2024.105875, PMID: 38685217

[ref52] Lockhart S. R. Chowdhary A. Gold J. A. W. (2023). The rapid emergence of antifungal-resistant human-pathogenic fungi. Nat. Rev. Microbiol. 21, 818–832. doi: 10.1038/s41579-023-00960-9 37648790 PMC10859884

[ref53] Lopez-Velasco G. Sbodio A. Tomas-Callejas A. Wei P. Tan K. H. Suslow T. V. (2012). Assessment of root uptake and systemic vine-transport of *Salmonella enterica* sv. *Typhimurium* by melon (*Cucumis melo*) during field production. Int. J. Food Microbiol. 158, 65–72. doi: 10.1016/j.ijfoodmicro.2012.07.005, PMID: 22824339

[ref54] Luo Y. Chen L. Lu Z. Zhang W. Liu W. Chen Y.. (2022). Genome sequencing of biocontrol strain *Bacillus amyloliquefaciens* Bam1 and further analysis of its heavy metal resistance mechanism. Bioresour. Bioprocess. 9:74. doi: 10.1186/s40643-022-00563-x, PMID: 38647608 PMC10991351

[ref55] Matilla M. A. Espinosa-Urgel M. Rodriguez-Herva J. J. Ramos J. L. Ramos-Gonzalez M. I. (2007). Genomic analysis reveals the major driving forces of bacterial life in the rhizosphere. Genome Biol. 8:R179. doi: 10.1186/gb-2007-8-9-r179, PMID: 17784941 PMC2375017

[ref56] Minh B. Q. Schmidt H. A. Chernomor O. Schrempf D. Woodhams M. D. von Haeseler A.. (2020). IQ-TREE 2: new models and efficient methods for phylogenetic inference in the genomic era. Mol. Biol. Evol. 37, 1530–1534. doi: 10.1093/molbev/msaa015, PMID: 32011700 PMC7182206

[ref57] Moore S. Vrebalov J. Payton P. Giovannoni J. (2002). Use of genomics tools to isolate key ripening genes and analyse fruit maturation in tomato. J. Exp. Bot. 53, 2023–2030. doi: 10.1093/jxb/erf057, PMID: 12324526

[ref58] Mora C. McKenzie T. Gaw I. M. Dean J. M. von Hammerstein H. Knudson T. A.. (2022). Over half of known human pathogenic diseases can be aggravated by climate change. Nat. Clim. Chang. 12, 869–875. doi: 10.1038/s41558-022-01426-1, PMID: 35968032 PMC9362357

[ref59] Nguyen N. H. Song Z. Bates S. T. Branco S. Tedersoo L. Menke J.. (2016). FUNGuild: An open annotation tool for parsing fungal community datasets by ecological guild. Fungal Ecol. 20, 241–248. doi: 10.1016/j.funeco.2015.06.006

[ref60] Olimi E. Kusstatscher P. Wicaksono W. A. Abdelfattah A. Cernava T. Berg G. (2022). Insights into the microbiome assembly during different growth stages and storage of strawberry plants. Environ Microbiome 17:21. doi: 10.1186/s40793-022-00415-3, PMID: 35484554 PMC9052558

[ref61] Pankievicz V. C. S. do Amaral F. P. Ane J.-M. Stacey G. (2021). Diazotrophic Bacteria and their mechanisms to interact and benefit cereals. Mol. Plant-Microbe Interact. 34, 491–498. doi: 10.1094/MPMI-11-20-0316-FI 33543986

[ref62] Parajuli A. Gronroos M. Siter N. Puhakka R. Vari H. K. Roslund M. I.. (2018). Urbanization reduces transfer of diverse environmental microbiota indoors. Front. Microbiol. 9:84. doi: 10.3389/fmicb.2018.00084, PMID: 29467728 PMC5808279

[ref63] Pastrana A. M. Borrero C. Perez A. G. Aviles M. (2023). Soilborne pathogens affect strawberry fruit flavor and quality. Plant Sci. 326:111533. doi: 10.1016/j.plantsci.2022.111533, PMID: 36375690

[ref64] Pathak H. K. Chauhan P. K. Seth C. S. Dubey G. Upadhyay S. K. (2024). Mechanistic and future prospects in rhizospheric engineering for agricultural contaminants removal, soil health restoration, and management of climate change stress. Sci. Total Environ. 927:172116. doi: 10.1016/j.scitotenv.2024.172116 38575037

[ref65] Ramli N. N. Othman A. R. Kurniawan S. B. Abdullah S. R. S. Hasan H. A. (2023). Metabolic pathway of Cr(VI) reduction by bacteria: a review. Microbiol. Res. 268:127288. doi: 10.1016/j.micres.2022.127288, PMID: 36571921

[ref66] Ranasinghe D. Lee E. S. Zhu Y. Frausto-Vicencio I. Choi W. Sun W.. (2019). Effectiveness of vegetation and sound wall-vegetation combination barriers on pollution dispersion from freeways under early morning conditions. Sci. Total Environ. 658, 1549–1558. doi: 10.1016/j.scitotenv.2018.12.159, PMID: 30678013 PMC7092696

[ref67] Robeson M. S. O'Rourke D. R. Kaehler B. D. Ziemski M. Dillon M. R. Foster J. T.. (2021). RESCRIPt: reproducible sequence taxonomy reference database management. PLoS Comput. Biol. 17:e1009581. doi: 10.1371/journal.pcbi.1009581, PMID: 34748542 PMC8601625

[ref68] Rognes T. Flouri T. Nichols B. Quince C. Mahe F. (2016). VSEARCH: a versatile open source tool for metagenomics. PeerJ 4:e2584. doi: 10.7717/peerj.2584, PMID: 27781170 PMC5075697

[ref69] Saini M. K. Capalash N. Varghese E. Kaur C. Singh S. P. (2020). Quantitative metabolomics approach reveals dynamics of primary metabolites in ‘Kinnow’ mandarin (*C. nobilis* × *C. deliciosa*) during advanced stages of fruit maturation under contrasting growing climates. J. Hortic. Sci. Biotechnol. 95, 106–112. doi: 10.1080/14620316.2019.1647118

[ref70] Savadiya B. Pandey G. Misra S. K. (2024). Remediation of pharmacophoric laboratory waste by using biodegradable carbon nanoparticles of bacterial biofilm origin. Environ. Res. 252:118969. doi: 10.1016/j.envres.2024.118969, PMID: 38642641

[ref71] Schaefer H. M. Ruxton G. D. (2011). “Evolutionary ecology of non-visual fruit traits” in Plant-animal communication. eds. Schaefer H. M. Ruxton G. D. (Oxford University Press).

[ref72] Schommer V. A. Vanin A. P. Nazari M. T. Ferrari V. Dettmer A. Colla L. M.. (2023). Biochar-immobilized Bacillus spp. for heavy metals bioremediation: a review on immobilization techniques, bioremediation mechanisms and effects on soil. Sci. Total Environ. 881:163385. doi: 10.1016/j.scitotenv.2023.163385, PMID: 37054796

[ref73] Scott R. A. Weil J. Le P. T. Williams P. Fray R. G. von Bodman S. B.. (2006). Long- and short-chain plant-produced bacterial N-acyl-homoserine lactones become components of phyllosphere, rhizosphere, and soil. Mol. Plant-Microbe Interact. 19, 227–239. doi: 10.1094/MPMI-19-0227, PMID: 16570653

[ref74] Shahraki A. Mohammadi-Sichani M. Ranjbar M. (2022). Identification of lead-resistant rhizobacteria of *Carthamus tinctorius* and their effects on lead absorption of sunflower. J. Appl. Microbiol. 132, 3073–3080. doi: 10.1111/jam.15410, PMID: 34897903

[ref75] Sharma M. Negi S. Kumar P. Srivastava D. K. Choudhary M. K. Irfan M. (2023). Fruit ripening under heat stress: the intriguing role of ethylene-mediated signaling. Plant Sci. 335:111820. doi: 10.1016/j.plantsci.2023.111820, PMID: 37549738

[ref76] Sheng L. Shen X. Su Y. Xue Y. Gao H. Mendoza M.. (2022). Effects of 1-methylcyclopropene and gaseous ozone on *Listeria innocua* survival and fruit quality of granny Smith apples during long-term commercial cold storage. Food Microbiol. 102:103922. doi: 10.1016/j.fm.2021.103922, PMID: 34809948

[ref77] Singh S. Singh S. Trivedi M. Dwivedi M. (2024). An insight into MDR *Acinetobacter baumannii* infection and its pathogenesis: potential therapeutic targets and challenges. Microb. Pathog. 192:106674. doi: 10.1016/j.micpath.2024.106674, PMID: 38714263

[ref78] Su J. Wang Y. Bai M. Peng T. Li H. Xu H. J.. (2023). Soil conditions and the plant microbiome boost the accumulation of monoterpenes in the fruit of *Citrus reticulata* 'Chachi'. Microbiome 11:61. doi: 10.1186/s40168-023-01504-2, PMID: 36973820 PMC10044787

[ref79] Thompson J. N. Willson M. F. (1979). Evolution of temperate fruit/bird interactions: Phenological strategies. Evolution 33, 973–982. doi: 10.2307/2407660, PMID: 28568428

[ref80] Tsitsigiannis D. I. Kunze S. Willis D. K. Feussner I. Keller N. P. (2005). *Aspergillus* infection inhibits the expression of peanut 13S-HPODE-forming seed lipoxygenases. Mol. Plant-Microbe Interact. 18, 1081–1089. doi: 10.1094/MPMI-18-1081, PMID: 16255247

[ref81] Wang B. Chen C. Xiao Y. M. Chen K. Y. Wang J. Zhao S.. (2024). Trophic relationships between protists and bacteria and fungi drive the biogeography of rhizosphere soil microbial community and impact plant physiological and ecological functions. Microbiol. Res. 280:127603. doi: 10.1016/j.micres.2024.127603, PMID: 38199002

[ref82] Wang X. Chi Y. Song S. (2024). Important soil microbiota's effects on plants and soils: a comprehensive 30-year systematic literature review. Front. Microbiol. 15:1347745. doi: 10.3389/fmicb.2024.1347745, PMID: 38591030 PMC10999704

[ref83] Wang X. Xu Q. Hu K. Wang G. Shi K. (2023). A coculture of *Enterobacter* and *Comamonas* species reduces cadmium accumulation in Rice. Mol. Plant-Microbe Interact. 36, 95–108. doi: 10.1094/MPMI-09-22-0186-R, PMID: 36366828

[ref84] Ward T. Larson J. Meulemans J. Hillmann B. Lynch J. Sidiropoulos D.. (2017). BugBase predicts organism level microbiome phenotypes. Oxford Academic.

[ref85] Whitehead S. R. Poveda K. (2011). Herbivore-induced changes in fruit—frugivore interactions. J. Ecol. 99, 964–969. doi: 10.1111/j.1365-2745.2011.01819.x

[ref86] Williams A. Sinanaj B. Hoysted G. A. (2024). Plant-microbe interactions through a lens: tales from the mycorrhizosphere. Ann. Bot. 133, 399–412. doi: 10.1093/aob/mcad191, PMID: 38085925 PMC11006548

[ref87] Wu F. Luo W. Li J. Xing W. Lyu L. Yang J.. (2022). Effects of arbuscular mycorrhizal fungi on accumulation and translocation of selenium in winter wheat. J. Sci. Food Agric. 102, 6481–6490. doi: 10.1002/jsfa.12015, PMID: 35570337

[ref88] Xi W. Zheng H. Zhang Q. Li W. (2016). Profiling taste and aroma compound metabolism during apricot fruit development and ripening. Int. J. Mol. Sci. 17:998. doi: 10.3390/ijms17070998, PMID: 27347931 PMC4964374

[ref89] Yang H. Fang C. Li Y. Wu Y. Fransson P. Rillig M. C.. (2022). Temporal complementarity between roots and mycorrhizal fungi drives wheat nitrogen use efficiency. New Phytol. 236, 1168–1181. doi: 10.1111/nph.18419 35927946

[ref90] Yiallouris A. Pana Z. D. Marangos G. Tzyrka I. Karanasios S. Georgiou I.. (2024). Fungal diversity in the soil mycobiome: implications for ONE health. One Health 18:100720. doi: 10.1016/j.onehlt.2024.100720, PMID: 38699438 PMC11064618

[ref91] Yin J. Yuan L. Huang J. (2021). New functions of *Ceriporia lacerata* HG2011: mobilization of soil nitrogen and phosphorus and enhancement of yield and quality of ketchup-processing tomato. J. Agric. Food Chem. 69, 4056–4063. doi: 10.1021/acs.jafc.0c06783, PMID: 33787254

[ref92] Yu X. Jiang N. Yang Y. Liu H. Gao X. Cheng L. (2023). Heavy metals remediation through bio-solidification: potential application in environmental geotechnics. Ecotoxicol. Environ. Saf. 263:115305. doi: 10.1016/j.ecoenv.2023.115305, PMID: 37517309

[ref93] Zhang R. Li S. Fu X. Pei C. Wang J. Wu Z.. (2022). Emissions and light absorption of PM(2.5)-bound nitrated aromatic compounds from on-road vehicle fleets. Environ. Pollut. 312:120070. doi: 10.1016/j.envpol.2022.120070 36058316

[ref94] Zhou Y. Coventry D. R. Gupta V. Fuentes D. Merchant A. Kaiser B. N.. (2020). The preceding root system drives the composition and function of the rhizosphere microbiome. Genome Biol. 21:89. doi: 10.1186/s13059-020-01999-0, PMID: 32252812 PMC7137527

[ref95] Zhou Y. Zhou S. (2023). Role of microplastics in microbial community structure and functions in urban soils. J. Hazard. Mater. 459:132141. doi: 10.1016/j.jhazmat.2023.132141, PMID: 37506647

[ref96] Zhu H. Gong L. Luo Y. Tang J. Ding Z. Li X. (2022). Effects of litter and root manipulations on soil bacterial and fungal community structure and function in a Schrenk’s spruce (*Picea schrenkiana*) Forest. Front. Plant Sci. 13:849483. doi: 10.3389/fpls.2022.849483, PMID: 35498706 PMC9047989

