# Supplementary material for: The influence of urban environmental effects on the orchard soil microbial community structure and function: a case study in Zhejiang, China
Source: Front Microbiol. 2024 Sep 9;15:1403443. doi: 10.3389/fmicb.2024.1403443 (PMC11417026; doi:10.3389/fmicb.2024.1403443)
Supplement: Supplementary file 1 [file Data_Sheet_1.docx]

**
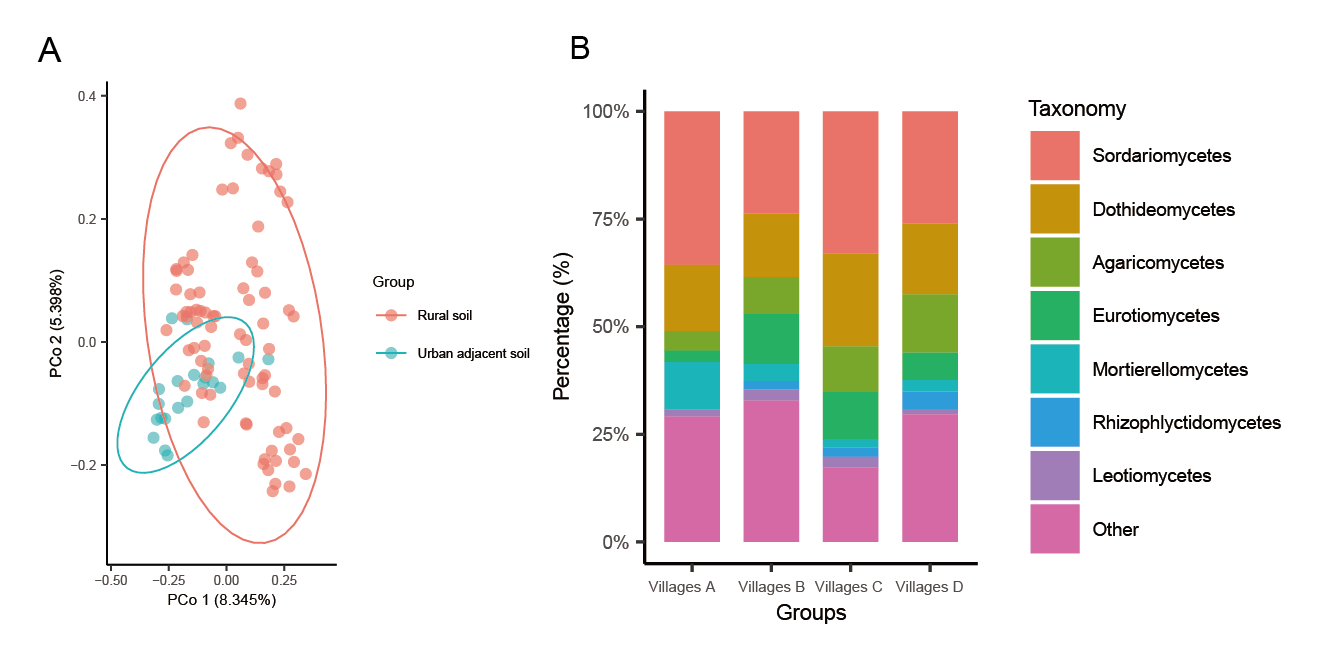
**

**Figure S1. Comparative analysis of fungal phenotypic differences between rhizospheric and peripheral soil. A**. PCoA analysis based on Bray-Curtis distances between pairs of fungal samples. The two dimensions with the highest explanatory power were plotted on the coordinate axes; **B**. Stacked bar chart showing the average abundance of fungal species at the class level in the four villages. The top 7 fungal species with the highest abundances were highlighted.


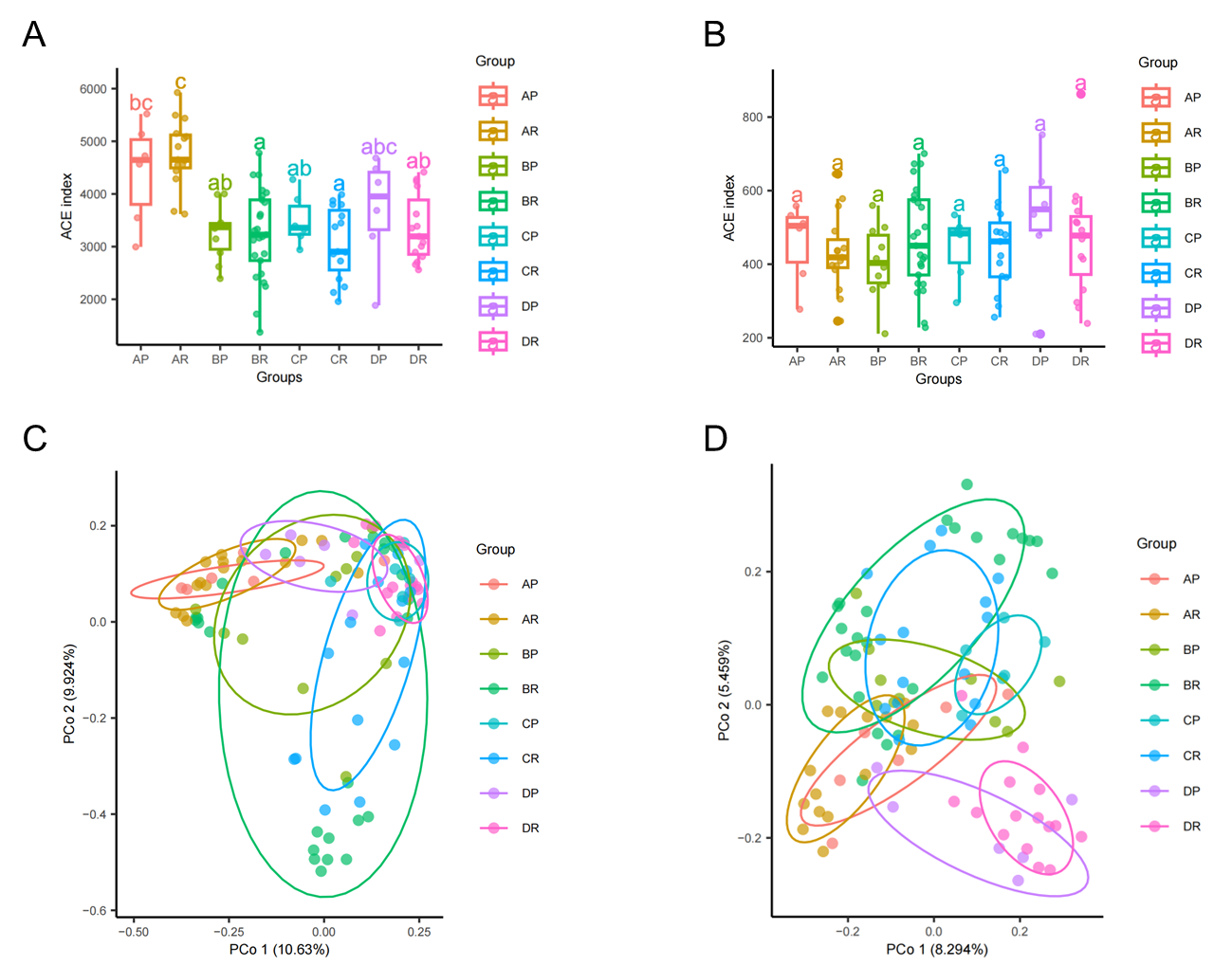
**Figure S2. Analyzing alpha and beta diversity to compare species composition variations across various villages based on rhizospheric and periphery soil.** **A & B**. The alpha diversity differences of bacteria (**A**) and fungi (**B**) among the four villages were assessed using the Abundance-based Coverage Estimator (ACE) index; Each point in the box plot represents the ACE index of a sample. The lowercase letters above the box plots indicate whether there is a statistical difference. The same letters indicate no statistical difference, while different letters indicate a statistical difference. **C & D**. PCoA analysis based on Bray-Curtis distances between pairs of bacterial (**C**) and fungal (**D**) samples. The two dimensions with the highest explanatory power were plotted on the coordinate axes. AR and AP represent the rhizospheric and peripheral samples, respectively, from village A. Similarly, BR and BP represent the rhizospheric and peripheral samples from village B, and so forth, up to village D.

**
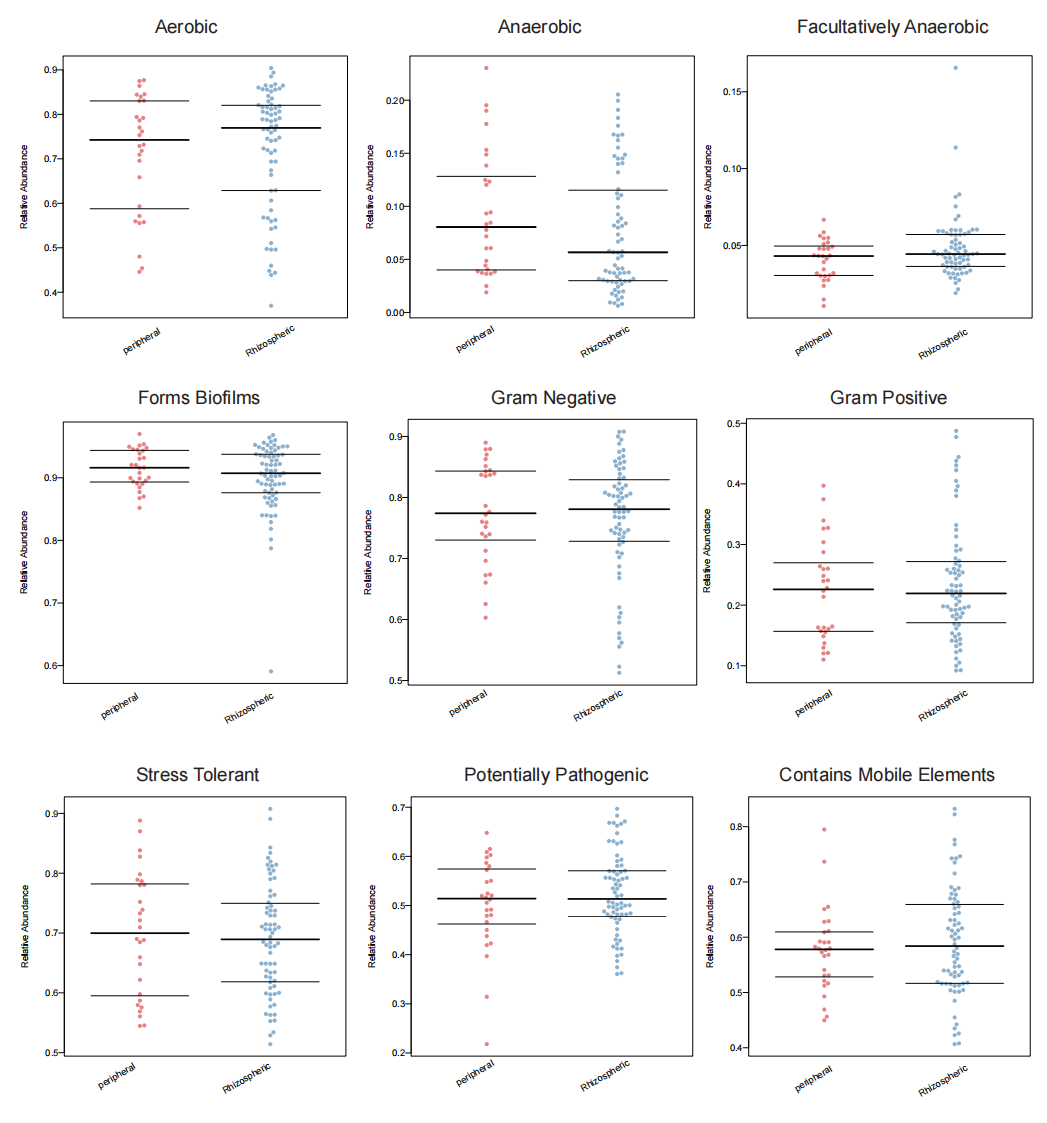
**

**Figure S3. Comparative bugbase phenotype analysis of rhizospheric and peripheral soil bacterial communities**

**
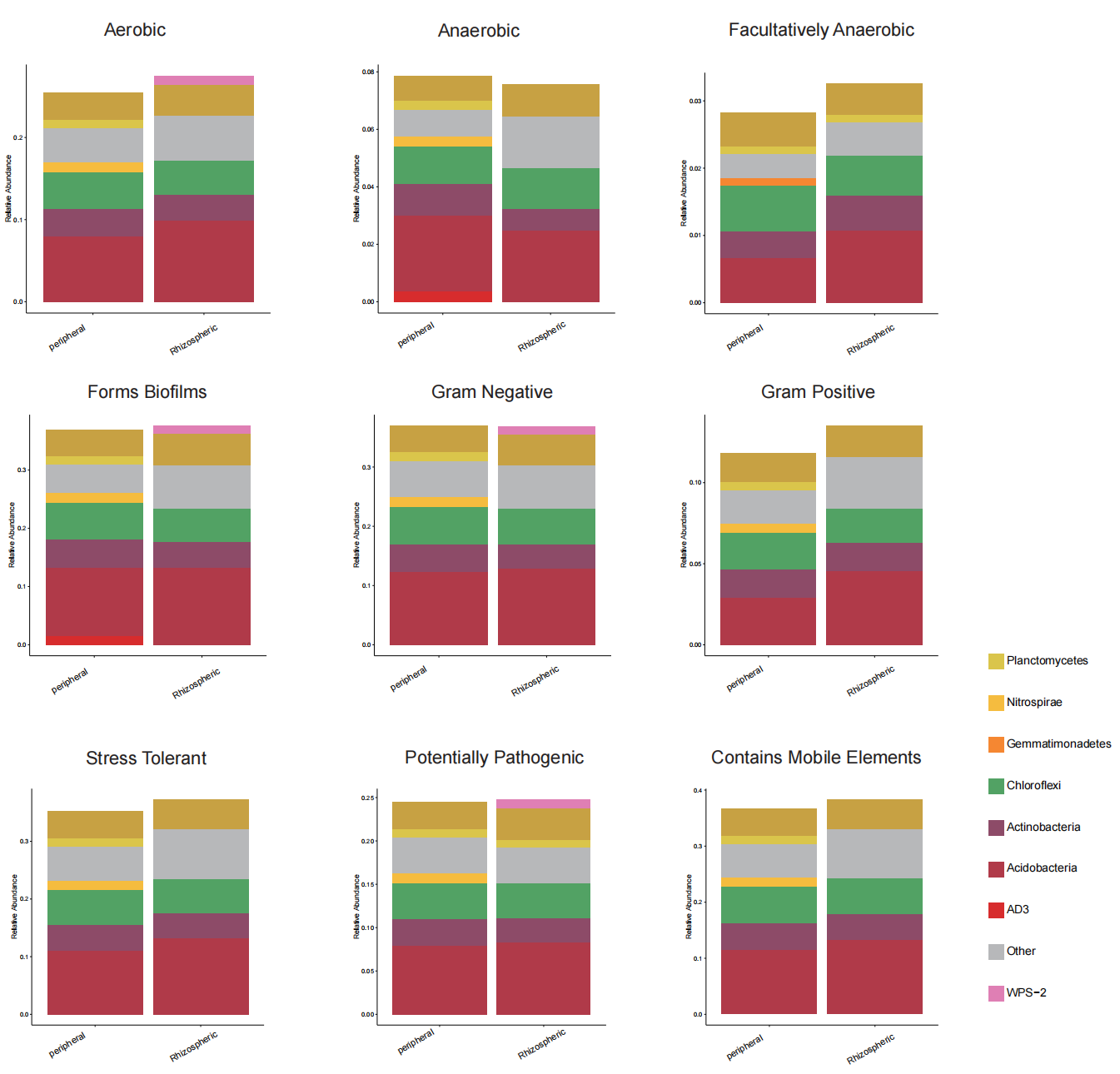
**

**Figure S4.** **Contribution of species to bugbase phenotypes in rhizospheric and peripheral soil bacterial communities**

**
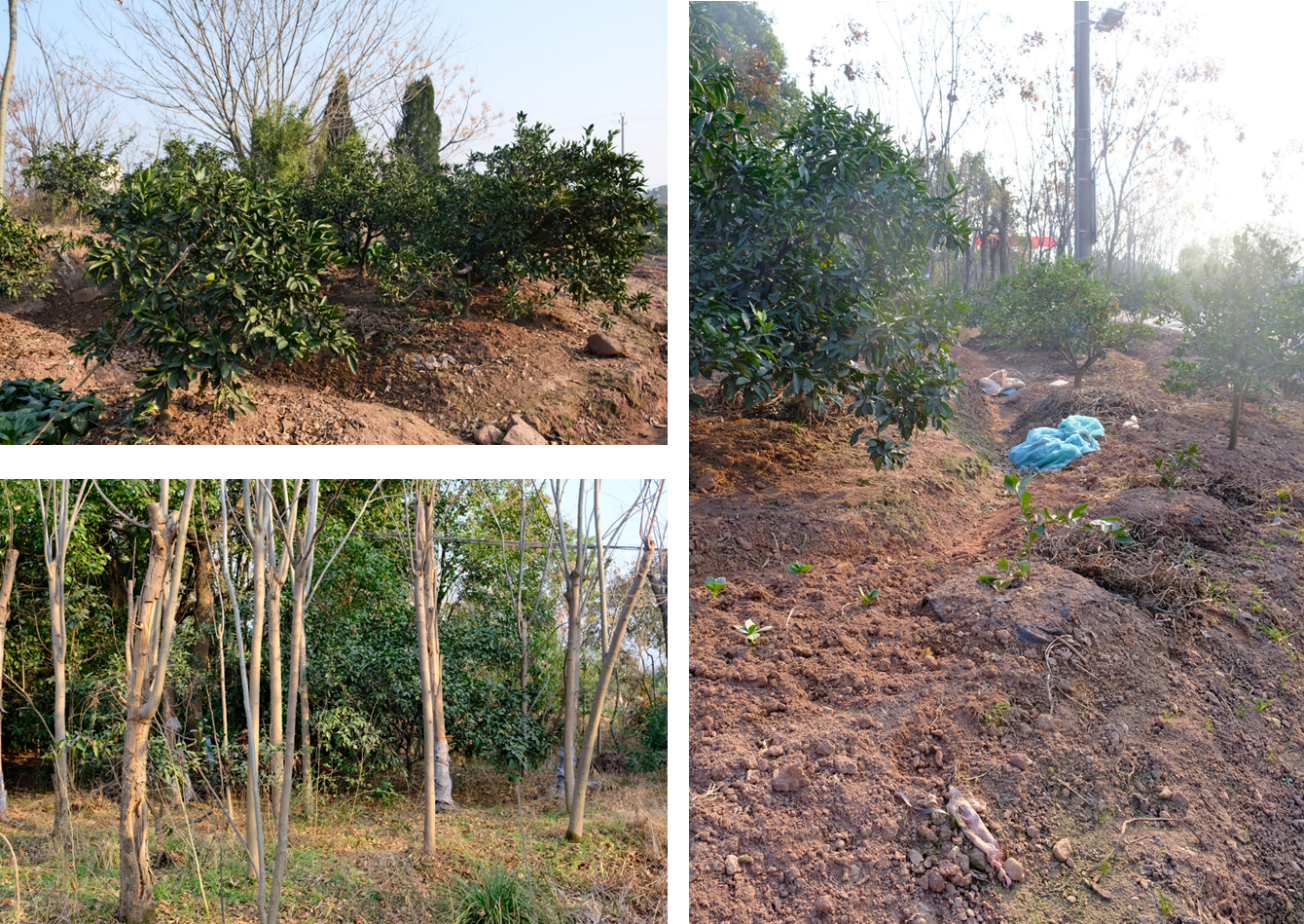
**

**Figure S5. Lack of managed citrus orchard soil environment after December harvest**
